# Polymorphism in Weberite Na_2_Fe_2_F_7_ and its Effects on Electrochemical Properties as a Na-Ion Cathode

**DOI:** 10.1021/acs.chemmater.3c00233

**Published:** 2023-04-25

**Authors:** Emily E. Foley, Vincent C. Wu, Wen Jin, Wei Cui, Eric Yoshida, Alexis Manche, Raphaële J. Clément

**Affiliations:** † Materials Department, University of California Santa Barbara, Santa Barbara, California 93106, United States; ‡ Materials Research Laboratory, University of California Santa Barbara, Santa Barbara, California 93106, United States; § Chemical Engineering Department, University of California Santa Barbara, Santa Barbara, California 93106, United States; ∥ Physics Department, University of California Santa Barbara, Santa Barbara, California 93106, United States

## Abstract

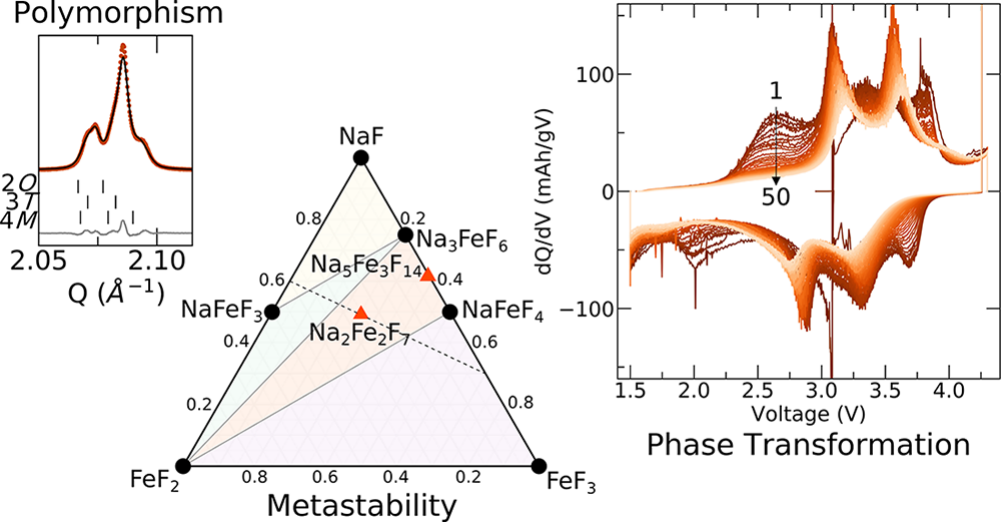

Weberite-type sodium transition metal fluorides (Na_2_*M*^2+^*M*′^3+^F_7_) have emerged as potential high-performance sodium intercalation cathodes, with predicted energy densities in the 600–800 W h/kg range and fast Na-ion transport. One of the few weberites that have been electrochemically tested is Na_2_Fe_2_F_7_, yet inconsistencies in its reported structure and electrochemical properties have hampered the establishment of clear structure–property relationships. In this study, we reconcile structural characteristics and electrochemical behavior using a combined experimental–computational approach. First-principles calculations reveal the inherent metastability of weberite-type phases, the close energetics of several Na_2_Fe_2_F_7_ weberite polymorphs, and their predicted (de)intercalation behavior. We find that the as-prepared Na_2_Fe_2_F_7_ samples inevitably contain a mixture of polymorphs, with local probes such as solid-state nuclear magnetic resonance (NMR) and Mössbauer spectroscopy providing unique insights into the distribution of Na and Fe local environments. Polymorphic Na_2_Fe_2_F_7_ exhibits a respectable initial capacity yet steady capacity fade, a consequence of the transformation of the Na_2_Fe_2_F_7_ weberite phases to the more stable perovskite-type NaFeF_3_ phase upon cycling, as revealed by *ex situ* synchrotron X-ray diffraction and solid-state NMR. Overall, these findings highlight the need for greater control over weberite polymorphism and phase stability through compositional tuning and synthesis optimization.

## Introduction

Batteries are found in nearly every facet of modern life, including in portable electronics, electric vehicles, and large-scale energy storage systems, largely enabled by lithium-ion batteries. However, with the imminent material supply shortage caused by the ever-increasing reliance on lithium (Li), nickel (Ni), and cobalt (Co) to produce Li-ion cathodes, more sustainable battery chemistries are needed. When considering possible electrode chemistries, sodium (Na)- and iron (Fe)-based compounds stand out as promising candidates. However, the higher atomic weight and larger ionic radius of Na^+^ compared to those of Li^+^ and the 0.3 V higher potential of the Na^+^/Na redox couple compared to that of Li^+^/Li lead to inherently lower theoretical energy densities for sodium-ion batteries (SIBs) compared to their Li-ion analogues. While significant work has gone toward developing layered oxide,^1−3^ polyanion,^4−6^ and Prussian blue^7,8^ cathodes for SIBs, viable Na alternatives to current Li systems have proven to be elusive.^9^ A paradigm shift in the development of competitive Na cathodes hinges on the investigation of new structural frameworks and anion chemistries.

Fluorine substitution has been shown to increase the operating voltage (and thereby the energy density) of both oxide^10,11^ and polyanion-based^12,13^ cathodes due to the inductive effect introduced by the highly electronegative F^–^ anion. However, pure transition metal fluorides are infrequently used as cathodes due to their generally poor ionic and electronic conduction properties.^14−17^ While electronic conduction can be partially mitigated by carbon coating, particle downsizing, and nanostructuring,^18−20^ ionic conduction is an intrinsic requirement for a topotactic Na (de)intercalation process. For poor ionic conductors, structure-preserving Na extraction and reinsertion processes are replaced by decomposition or conversion reactions. Conversion reactions should in theory result in high capacities but are poorly reversible in practice:^21,22^ they have been reported for cryolite-like Na_3_*M*F_6_^23^ and rutile *M*F_2_^24,25^ and for perovskite-type *M*F_3_^14,26,27^ and Na*M*F_3_^28,29^ on deep discharge. Clearly, transition metal fluoride structures containing fast Na-ion transport pathways are needed. In this regard, weberite compounds (with general formula Na_2_*M*^2+^*M*′^3+^F_7_) have been shown to exhibit an open framework structure that holds promise for facile Na-ion diffusion and topotactic Na-ion (de)intercalation.^30−32^

Weberite-type sodium transition metal fluorides have been studied for their magnetic properties since the 1970s^33,34^ but have only been explored as Na-ion battery electrode materials over the past few years.^30−32,35,36^ In 2019, Euchner *et al.* predicted, using first-principles calculations, that this material class should lead to good Na-ion diffusion properties, high Na insertion potentials, and high capacities, resulting in energy densities competitive with those of common Li-ion cathode materials.^35^ A recent experimental report by Park *et al.* demonstrated exceptional performance for the Na_2_Fe_2_F_7_ weberite cathode, including an initial capacity of 184 mAh/g at C/20 and an outstanding full cell capacity retention of 88.3% at 2C (initial capacity of 118 mAh/g) after 1000 cycles.^30^ While Na_2_Fe_2_F_7_ remains the best-performing weberite cathode to date,^30^ other chemistries such as Na_2_TiFeF_7_^31^ and Na_2_*M*VF_7_ (*M* = Mn, Fe, and Co)^32^ have been shown to result in capacities in excess of 185 and 147 mAh/g, respectively.

While Park *et al.* reported a trigonal Na_2_Fe_2_F_7_ weberite,^30^ two other variants of the weberite structure (orthorhombic and monoclinic) have been reported for this compound,^36,37^ indicating a complex phase stability landscape for Na_2_Fe_2_F_7_, with direct impacts on its applicability as an intercalation electrode. Here, we report an in-depth computational–experimental investigation of the structure, phase stability, and electrochemistry of Na_2_Fe_2_F_7_, aiming to establish clear links between local and long-range structural features and Na intercalation properties. We find that Na_2_Fe_2_F_7_ is prone to polymorphism, with all three weberite forms present in our samples despite extensive synthesis optimization, as evidenced by synchrotron X-ray diffraction (SXRD) and supported by ^57^Fe Mössbauer and ^23^Na solid-state nuclear magnetic resonance (ss-NMR) spectroscopy. Notably, this is the first NMR investigation of a weberite material (supplemented with first-principles calculations of NMR parameters) and provides key insights into the Na local environments. From an electrochemical performance standpoint, we highlight both the promise presented and difficulties faced by weberite cathodes such as Na_2_Fe_2_F_7_. While density functional theory (DFT) calculations reveal that all three Na_2_Fe_2_F_7_ polymorphs are capable of exchanging 2 Na ions per formula unit (compositions between NaFe_2_F_7_ and Na_3_Fe_2_F_7_) through a solid-solution mechanism on charge and discharge, kinetic limitations on deep discharge limit the practical capacity, which may be related to the large predicted volume expansion at high Na contents. We further identify a phase transformation upon cycling, revealed by *ex situ* SXRD and ^23^Na ss-NMR. While this study confirms the suitability of weberites as potential high-energy cathode materials for SIBs, it also highlights a need for controlled synthesis routes to obtain single-phase weberite samples, for the optimization of electrode preparation methods to achieve the full redox capacity of Na_2_Fe_2_F_7_ and related systems and for a better understanding of the metastability issues facing this class of cathode materials.

## Results and Discussion

### Weberite Structure and its Variants

**Figure 1:**
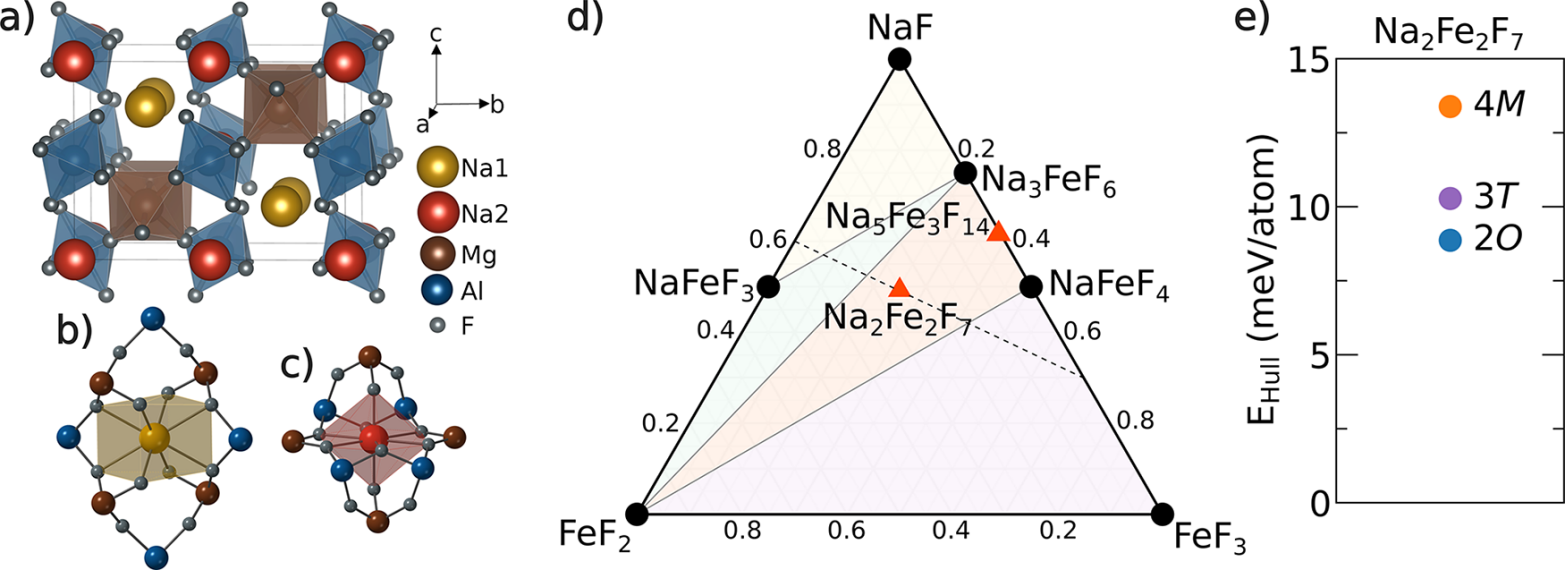
Weberite structure, stability, and polymorphism. Crystal structure of the Na_2_MgAlF_7_ weberite mineral, highlighting the *M*F_6_ (where *M* = Mg, Al) polyhedral network in (a) and the local Na coordination environments: Na1 in distorted cubic sites in (b) and Na2 in bihexagonal pyramidal sites in (c). (d) DFT-predicted NaF-FeF_2_-FeF_3_ ternary phase diagram, where stable compounds are shown as black circles, metastable compounds as orange triangles, and regions of phase coexistence are shaded. The black, dotted tie line represents the compositional evolution of the weberite at various stages of charge. (e) Energy above the hull for the three Na_2_Fe_2_F_7_ weberite variants.

The weberite structure is a fluorine-deficient superstructure of fluorite (CaF_2_) with general formula *A*_2_*B*_2_*X*_7_, where *A* and *B* are cations and *X* is an anion. The high-symmetry variant (*Imma* space group) and *A*-site local environments are shown in Figure 1a–c for the weberite mineral, Na_2_MgAlF_7_. Weberites retain the same cationic face-centered cubic stacking sequence as fluorite, but as they are anion-deficient, the *B* cations are now only 6-fold coordinated, while the *A* cations retain the 8-fold coordination typical of the fluorite structure. The mixed-valence *B* cations, *B*1 and *B*2 (typically divalent *M*^2+^ and trivalent *M*^3+^ metals, respectively), form a network of three-dimensional corner-connected octahedra composed of one-dimensional chains of *B*1 octahedra that are connected to each other by *B*2 octahedra. The *A* cations occupy distorted cubic sites (*A*1, Figure 1b) and bihexagonal pyramidal sites (*A*2, Figure 1c). The *A*1 polyhedra form edge-sharing chains that also share edges with the *A*2 corner-connected polyhedral chains, creating a three-dimensionally connected *A* cation network. Additionally, the weberite structure can be viewed as alternating close-packed cation layers of *A*_3_*B* and *AB*_3_, as shown in Figure S1. In each layer, the majority cation species (*e.g.*, *A* in *A*_3_*B*) forms a Kagomé-type network, and the minority cation species occupies the center of the Kagomé rings.

Several weberite variants have been reported that differ in their stacking sequence of the Kagomé-like layers. A nomenclature system has been proposed by Grey *et al.*(38) based on the crystal system and number of close-packed cation slabs (*e.g.*, a pair of *A*_3_*B* and *AB*_3_ layers) within the unit cell. For example, the high-symmetry weberite variant (shown in Figure 1a) is denoted 2*O* as it is based on the orthorhombic crystal system, and the smallest repeating unit contains two cation slabs. Na-*M*-F weberites reported to date form the 2*O* (*Imma*), 2*M* (monoclinic *C*2/*c*), 3*T* (trigonal *P*3_1_21), and/or 4*M* (monoclinic *C*2/*c*) structures, where the different slab stackings can be summarized as *AA* for 2*O*, *ABAB* for 2*M*, *AABBAABB* for 4*M*, and *ABCABC* for 3*T*. Structural diagrams that illustrate those differences are shown in Figure S2. We note that the 2*M* variant has only been reported for Na_2_CuGaF_7_, and we do not consider this variant further in this work.

### Na_2_Fe_2_F_7_ Polymorphism and Energetics

Given the structural diversity of weberites, a detailed investigation of possible Na_2_Fe_2_F_7_ polymorphism and its impact on electrochemical properties is warranted if this material is to be considered for SIB applications. In 1993, a single-crystal XRD study by Yakubovich *et al.* suggested that Na_2_Fe_2_F_7_ crystallizes in the 4*M* weberite variant,^37^ in line with prior reports of the 4*M* structure for other Fe^2+^-based weberites.^33,39,40^ Yet, more recently, Dey *et al.*(36) and Park *et al.*(30) reported the synthesis of the 2*O* and 3*T* variants of Na_2_Fe_2_F_7_, respectively, and their electrochemical properties in Na-ion cells. These contrasting reports are difficult to reconcile, especially since they either do not include any diffraction data,^37^ or they include patterns with significantly broadened reflections,^30,36^ and that lack both the sensitivity and resolution to distinguish between the very similar diffraction patterns of the 4*M*, 2*O*, and 3*T* polymorphs (see simulated patterns in Figure S3). The main differences between those patterns lie in the splitting of select peaks and a few low-intensity peaks characteristic of the 3*T* and 4*M* structures. Thus, in order to identify the weberite structural variant(s) present in a given sample using XRD, high-quality data on highly crystalline materials—if not single crystals—is necessary.

To gain insights into the likelihood of obtaining the various weberite structural variants of Na_2_Fe_2_F_7_, the phase stability of Na-Fe-Fe compounds was investigated. The formation energy of each polymorph and competing phases was calculated using DFT, with the resultant NaF-FeF_2_-FeF_3_ ternary diagram shown in Figure 1d (values in Table S1). Within this space, the weberite polymorphs and Na_5_Fe_3_F_14_ are metastable, lying 5 to 15 meV/atom above the hull, while the most stable phase is Na_3_FeF_6_, lying on the hull. As illustrated in Figure 1e, the energy above the hull for each weberite polymorph is within 5 meV/atom of each other, suggesting that all three of the polymorphs may be stabilized at room temperature. These DFT results and the multiple polymorphs previously reported for Na_2_Fe_2_F_7_ suggest that XRD patterns for Na_2_Fe_2_F_7_ weberites should be analyzed with care and complemented with local structure characterization to identify the polymorph(s) present in a given sample.

### Synthesis and Long-Range Structural Characterization of Na_2_Fe_2_F_7_

**Figure 2:**
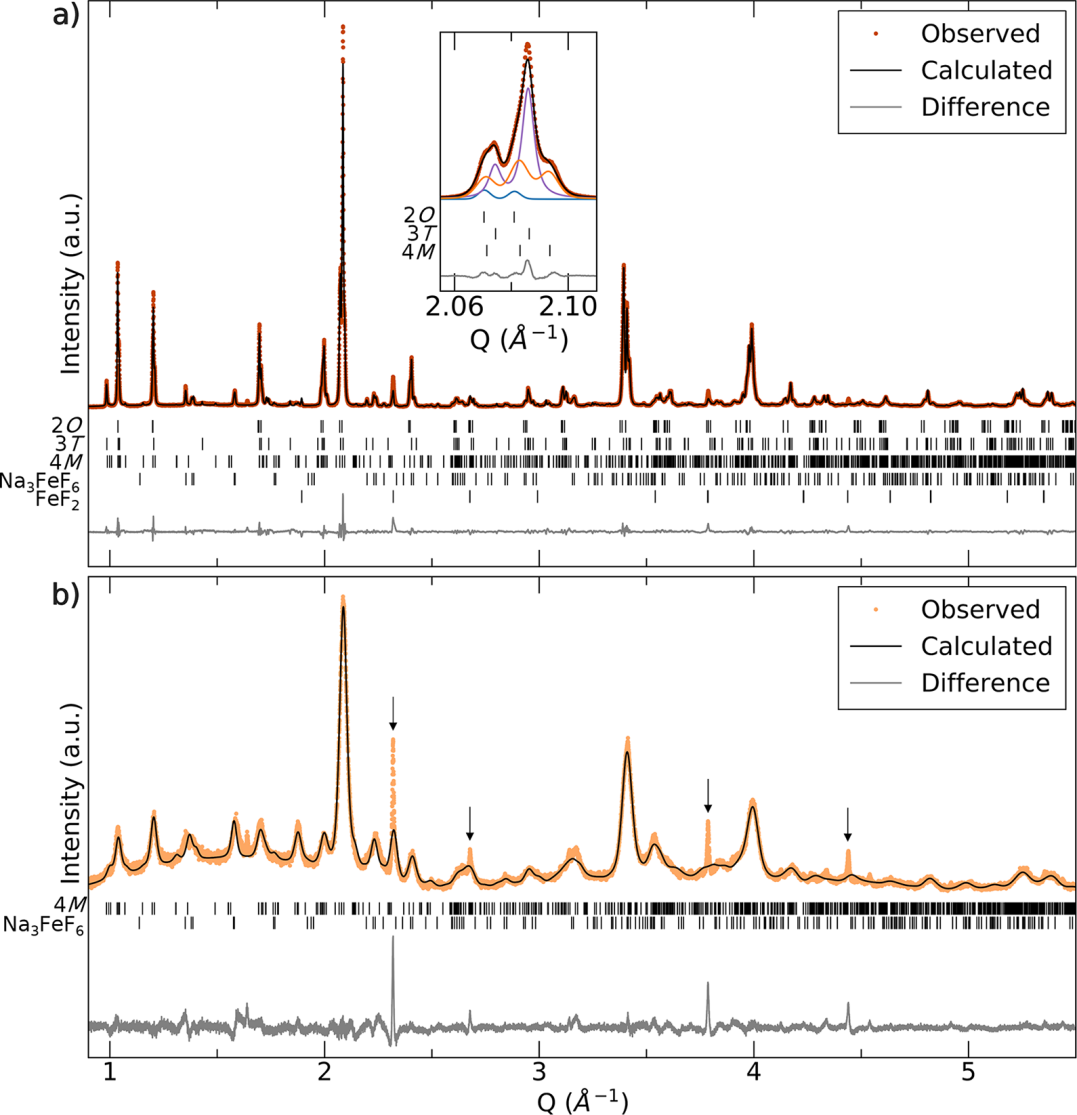
Synchrotron XRD characterization of Na_2_Fe_2_F_7_ weberite samples. Synchrotron XRD pattern collected on (a) pristine and (b) carbon-coated Na_2_Fe_2_F_7_ with their corresponding Rietveld refinements. The inset in (a) shows an enlarged version of the refinement results for the main weberite peaks, with individual profiles shown for the 2*O* (blue), 3*T* (purple), and 4*M* (orange) polymorphs. The arrows in (b) indicate reflections associated with an unidentified impurity phase.

Na_2_Fe_2_F_7_ was prepared using a mechanochemically assisted solid-state synthesis procedure adapted from Park *et al.*(30) Several annealing temperatures were tested, and the laboratory powder diffraction patterns obtained on the as-prepared samples are shown in Figure S4. While Park *et al.* utilized an annealing temperature of 650 °C, we found that this temperature resulted in a large amount of impurities (namely, Na_3_FeF_6_ and FeF_2_). In contrast, an annealing temperature of 500 °C minimized the amount of impurity phases and was used for the remainder of this study. The SXRD pattern collected on the 500 °C annealed sample and its corresponding Rietveld refinement are shown in Figure 2a. Despite careful optimization of the synthesis conditions, impurity phases were still observed (9.5 wt % Na_3_FeF_6_ and 0.9 wt % FeF_2_). The presence of a significant amount of Na_3_FeF_6_ in our sample can be explained by its very negative formation energy (Figure 1d) compared to that of the weberite Na_2_Fe_2_F_7_ variants.

The SXRD data collected on Na_2_Fe_2_F_7_ was analyzed using a combination of Pawley and Rietveld refinements. Refinements involving a single weberite polymorph resulted in poor fits (χ^2^ > 3.5, *R*_WP_ > 17%) due to the complexity of the experimental peak shapes (Figure 2a inset), clearly suggesting the presence of many overlapping diffraction peaks and a mixture of phases. Refinement models using all three weberite polymorphs resulted in much improved refinements, with the best refinement (χ^2^ = 2.12, *R*_WP_ = 9.75%) indicating a sample composition of 4.4 wt % of 2*O*, 42.4 wt % of 3*T*, and 42.8 wt % of the 4*M* polymorph. To prevent overfitting the diffraction pattern, site occupancies were not refined, and thermal factors were constrained to be equal for each atom type, with detailed Rietveld refinement parameters listed in Tables S2 and S3. An additional refinement was performed without the 2*O* polymorph (Figure S5), which resulted in a moderately worse fit (χ^2^ = 2.16, *R*_WP_ = 9.94%), indicating that the 2*O* polymorph is in fact present. Given that a multi-phasic Na_2_Fe_2_F_7_ sample was obtained in this work, consistent with the similar energetics of the various Na_2_Fe_2_F_7_ polymorphs obtained from first principles (Figure 1c), it is possible that previous studies of this material overlooked the presence of multiple phases in their samples.

**Figure 3:**
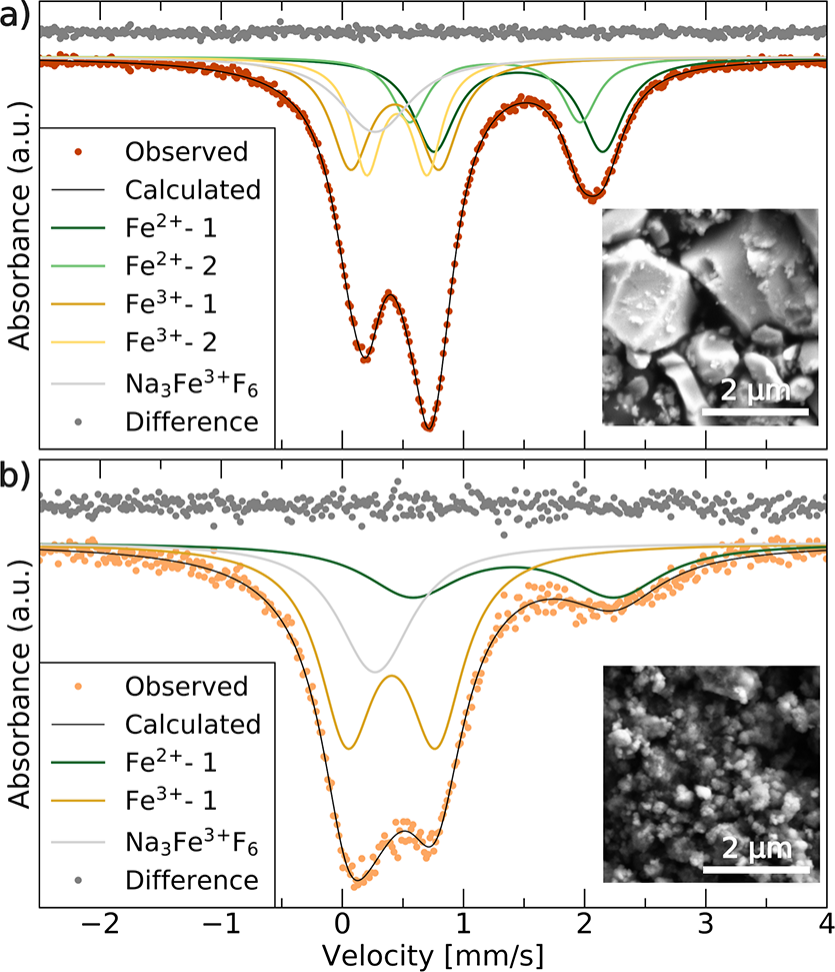
^57^Fe Mössbauer spectroscopy and SEM characterization of Na_2_Fe_2_F_7_ weberite samples. (a) ^57^Fe Mössbauer spectrum and fit collected on (a) pristine and (b) carbon-coated Na_2_Fe_2_F_7_. The insets show the corresponding SEM micrographs for each material.

In preparation for electrochemical testing, the pristine material was ball-milled with carbon to improve its electronic conductivity through both particle size reduction and carbon coating.^19,41,42^ Scanning electron microscopy (SEM) images are shown in the Figure 3a,b inset for pristine and carbon-coated Na_2_Fe_2_F_7_. The pristine Na_2_Fe_2_F_7_ sample contains ≈1–2 μm particles, while the carbon-coated Na_2_Fe_2_F_7_ sample contains ≈100 nm particles, indicating successful particle downsizing. The SXRD pattern collected on carbon-coated Na_2_Fe_2_F_7_, shown in Figure 2b, exhibits significant peak broadening and a poor signal-to-noise ratio, a likely consequence of the small average particle size and the buildup of strain in the material during the ball-milling process. Thus, the sole purpose of the Rietveld refinement was to identify the amount of impurity phases present: this was done using a single weberite phase, 4*M*, with detailed Rietveld refinement parameters listed in Table S4. The amount of impurity phases in the carbon-coated sample is similar to that prior to carbon coating but with 11.4 wt % of Na_3_FeF_6_ and no FeF_2_ present. The SXRD pattern collected on carbon-coated Na_2_Fe_2_F_7_ also exhibits a series of low-intensity, sharp reflections indicated with arrows in Figure 2b and likely arising from a minor, high-symmetry impurity phase. Despite an extensive search comprising all known binary or ternary phases containing the elements Na, Fe, F, C, Zr, and/or O (with Zr and O arising from the ZrO_2_ ball mill jar used here), we are unable to assign those reflections, which cannot be fit using an anisotropic strain model.^43^

### Insights into Fe and Na Local Environments from Mössbauer and Solid-State NMR Spectroscopy

^57^Fe Mössbauer spectroscopy was used to characterize the Fe local environments in the pristine and carbon-coated Na_2_Fe_2_F_7_ samples. The spectrum collected on pristine Na_2_Fe_2_F_7_ is shown in Figure 3a. A good fit (χ^2^ = 1.12) of this spectrum was obtained using five components (fitting parameters are provided in Table S5): two doublets at isomer shifts (δ) of 1.45 and 1.25 mm/s, with quadrupolar splittings (Δ*E*_Q_) of 1.38 and 1.42 mm/s, respectively, assigned to Fe^2+^F_6_ in the weberite phases; two doublets at δ = 0.43 and 0.45 mm/s, with Δ*E*_Q_ = 0.72 and 0.49 mm/s, respectively, assigned to Fe^3+^F_6_ in the weberite phases; and a doublet assigned to Na_3_FeF_6_ using previously reported Mössbauer parameters (δ = 0.27 mm/s, Δ*E*_Q_ = 0.15 mm/s).^23^ The Fe^2+^F_6_ and Fe^3+^F_6_ signals closely match values obtained for other Fe-containing weberite phases.^36,44−46^ The multiple doublets assigned to Fe^2+^F_6_ and to Fe^3+^F_6_ species indicate a distribution of Fe local environments in the distinct weberite polymorphs. Notably, the Fe^2+^F_6_ local environment changes more than the Fe^3+^F_6_ local environment in the various forms of Na_2_Fe_2_F_7_, which may account for the larger variation in the isomer shift of Fe^2+^F_6_ signals. Integration of the Mössbauer signals leads to an average Fe oxidation state of 2.55+ for the weberite phases resulting in a stoichiometry of Na_1.90_Fe^2.55+^_2_F_7_ (assuming full F occupation)^47^ indicating a slight Na deficiency, in line with the inductively coupled plasma (ICP) results (Table S6).

The ^57^Fe Mössbauer spectrum collected on the carbon-coated Na_2_Fe_2_F_7_ sample (Figure 3b) suggests moderate Fe oxidation upon carbon coating (average Fe oxidation state of 2.67+). Notably, this spectrum could be fitted with one broad signal for Fe^2+^F_6_ and one broad signal for Fe^3+^F_6_ (fitting parameters are provided in Table S5), indicating greater disorder within the weberite phase(s) upon ball-milling. Overall ^57^Fe Mössbauer spectroscopy confirms that the weberite material is slightly Na-deficient, with this deficiency increasing upon carbon coating, resulting in a stoichiometry of about Na_1.68_Fe^2.7+^_2_F_7_ prior to electrochemical cycling.

The Na local environments in Na_2_Fe_2_F_7_ were examined using ^23^Na ss-NMR. As Na_2_Fe_2_F_7_ contains unpaired electrons from the Fe^2+^/Fe^3+^ species and ^23^Na is a quadrupolar nucleus (*I* = 3/2), both paramagnetic and quadrupolar effects are present. These interactions result in significant NMR line broadening and large chemical shifts, leading to significant signal overlap and complicating the attribution of spectral features to specific local environments in the material. Here, the assignment of the complex NMR spectra was assisted by first-principles hybrid DFT/Hartree–Fock (HF) calculations of ^23^Na NMR parameters using the CRYSTAL17 code on the different Na_2_Fe_2_F_7_ weberite structural variants (see Note S1 for the computational parameters and methodology), with results shown in Table S7. The ^23^Na isotropic chemical shift (δ_iso_) is dominated by the paramagnetic (Fermi contact) shift resulting from delocalization of unpaired electron spin density from nearby Fe 3d orbitals to the ^23^Na s orbital. These paramagnetic interactions also lead to significant spectral broadening. The interaction between the ^23^Na nuclear quadrupole moment and the electric field gradient present at the Na nucleus leads to a further broadening of the spectrum and to a shift of the ^23^Na resonant frequency due to second-order effects (denoted δ_Q_). The observed chemical shift (δ_obs_) is then the sum of the isotropic Fermi contact shift and of the second-order quadrupolar shift: δ_obs_ = δ_iso_ + δ_Q_.

**Figure 4:**
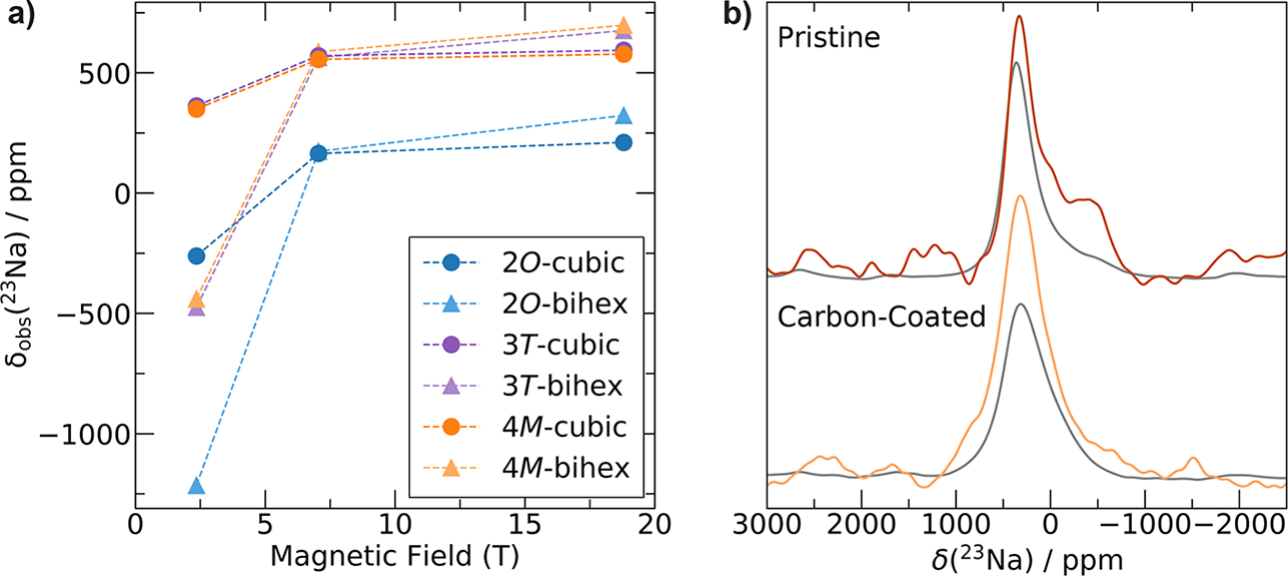
(a) Field dependence of the calculated chemical shifts for the average cubic and bihexagonal pyramidal sites in Na_2_Fe_2_F_7_ weberite polymorphs. (b) ^23^Na ss-NMR spin-echo spectra collected on pristine and carbon-coated Na_2_Fe_2_F_7_ using π/2 (gray) and π/6 (orange) excitation pulses.

Obtaining high-resolution spectra of quadrupolar nuclei in paramagnetic materials is complicated by the opposing magnetic field (*B*_0_) dependence of quadrupolar and paramagnetic interactions. While second-order quadrupolar effects (*e.g.*, δ_Q_ and associated line broadening) are inversely related to *B*_0_, such that NMR spectra of quadrupolar nuclei are typically obtained in high fields, high field strengths exacerbate paramagnetic broadening, and lower fields are preferred for strongly paramagnetic samples. Here, the optimal field strength that maximizes spectral resolution was determined by plotting the predicted δ_obs_ at various magnetic field strengths for cubic and bihexagonal pyramidal Na sites in each weberite polymorph, as shown in Figure 4a. The highly asymmetrical bihexagonal pyramidal Na environments exhibit a greater field dependence of their δ_obs_ due to their larger quadrupolar coupling constants (*C*_Q_ ≥ 5 MHz) compared to cubic Na sites (*C*_Q_ ≤ 3.5 MHz). Our first-principles calculations suggest the following: (1) significant overlap between the ^23^Na ss-NMR signals associated with cubic Na sites in the 3*T* and 4*M* weberite variants and similarly for the bihexagonal pyramidal Na signals in these two phases. For the 2*O* polymorph, the resonance associated with the cubic Na environment likely overlaps with that of the bihexagonal pyramidal Na signals from the 3*T* and 4*M* weberite variants, while the signal arising from 2*O* bihexagonal pyramidal Na sites is expected at very negative shifts and should be discernible. (2) At low fields (2.35 T), ^23^Na signals corresponding to cubic and bihexagonal pyramidal Na sites within a single weberite phase are expected to be separated by nearly 1000 ppm owing to their different second-order quadrupolar shifts (δ_Q_), while at higher fields, δ_Q_ becomes negligible, and the signals overlap. Thus, to maximally resolve ^23^Na signals from the different local environments present in the samples of interest, all ^23^Na ss-NMR spectra were obtained in our lowest field of 2.35 T.

^23^Na ss-NMR spin-echo spectra collected on pristine and carbon-coated Na_2_Fe_2_F_7_ samples are shown in Figure 4b. For each sample, the two overlaid spectra were obtained using a typical π/2 excitation pulse and a small flip angle (π/6) excitation pulse, respectively. Despite the decrease in sensitivity caused by the small flip angle, the π/6 spectra exhibit at least one additional signal at ≈ −400 ppm compared to the π/2 spectra that is more prevalent for pristine Na_2_Fe_2_F_7_. This additional signal is attributed to bihexagonal pyramidal Na sites, whose larger quadrupolar interactions result in broader, lower-intensity signals that are poorly excited by a π/2 pulse.^48^ Thus, the π/2 spectra are dominated by signals arising from Na in cubic sites, while the π/6 spectra reflect all Na environments present in the samples. The spectra also contain low-intensity signals arising from the Na_3_FeF_6_ impurity phase, as revealed by the ^23^Na ss-NMR spin-echo spectrum obtained on a Na_3_FeF_6_ sample overlaid in Figure S6 with the π/2 spectra shown in Figure 4b. While the 200 ppm Na_3_FeF_6_ signal is difficult to discern from the Na_2_Fe_2_F_7_ phase, the Na_3_FeF_6_ signal at 1800 ppm is distinguishable but very low in intensity. Finally, while the π/6 spectra shown in Figure 4b could in theory provide some information on the relative population of cubic and bihexagonal pyramidal Na sites, the strong quadrupolar and paramagnetic interactions present in Na_2_Fe_2_F_7_ result in short ^23^Na ss-NMR signal lifetimes and signal loss over the course of the experiments. Hence, the relative signal intensities in these spectra are not quantitative; besides, measurements of transverse relaxation times (*T*_2_′) to account for signal loss are impractical due to their extremely low sensitivity (*e.g.*, the π/6 spectrum collected on pristine Na_2_Fe_2_F_7_ took over 48 h to be acquired).

Nearly all the ^23^Na ss-NMR signals from the weberite variants are predicted to lie within the −500–500 ppm range, save from the bihexagonal pyramidal Na resonance and the Na signals arising from the 2*O* variant that exhibit more negative shifts (see Figure 4a). Clearly, all the main resonances observed in the spectra shown in Figure 4b lie within the expected range for Na_2_Fe_2_F_7_ weberites. Yet, the π/6 spectra show clear differences in the distribution of Na local environments in the pristine and carbon-coated Na_2_Fe_2_F_7_ samples. The spectrum collected on the pristine sample exhibits signals at negative shifts (centered around −400 ppm) consistent with the presence of Na in bihexagonal pyramidal sites in the 3*T* and 4*M* phases. Notably, this spectrum appears to contain several overlapping signals, including distinct cubic and bihexagonal pyramidal signals, suggestive of multiple polymorphs being present. The carbon-coated sample, however, shows noticeably fewer signals at negative shifts, suggesting that the C-coating process may be reducing the intensity of the quadrupolar interactions and/or reducing the occupation of bihexagonal pyramidal Na sites by introducing more overall disorder into the system.

These results, combined with the previously discussed SXRD and ^57^Fe Mössbauer data, suggest the preparation of a multi-phasic (predominantly 3*T* and 4*M*) Na_2_Fe_2_F_7_ weberite material containing Na_3_FeF_6_ impurities that are partially disordered upon carbon coating while still retaining the long-range weberite structure.

### Electrochemical Properties of Na_2_Fe_2_F_7_

As the 24 h carbon-coating process affects the weberite structure, several shorter mechanochemical milling times and an *in situ* carbon-coating method were investigated to reduce structural disordering, with results presented in Note S2. Briefly, none of the alternative carbon-coating methods tested here led to an electrochemical performance on par with that obtained after a 24 h milling step, which was therefore adopted for the remainder of this work.

**Figure 5:**
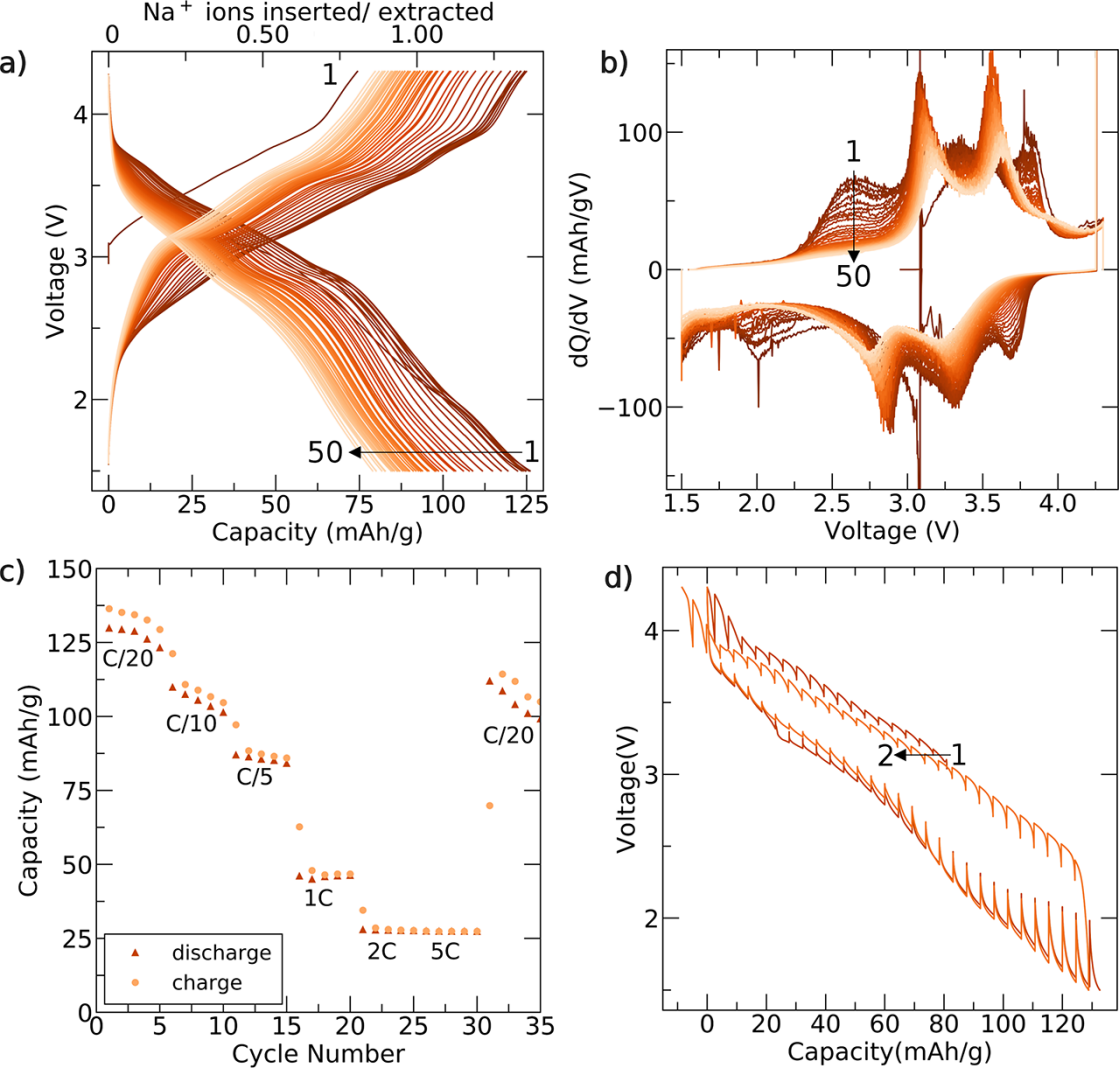
Electrochemical characterization of Na_2_Fe_2_F_7_. (a) Galvanostatic charge–discharge curves recorded at C/20 over the first 50 cycles and (b) corresponding differential capacity plots. (c) Charge and discharge capacity obtained at various cycling rates and recorded over 35 cycles. The first charge step was omitted from the plot for clarity. (d) GITT data obtained over the first 2 cycles, where a 30 min C/20 current pulse was applied followed by a 2 h rest period. All the electrochemical results shown here were obtained using voltage cutoffs of 4.3 and 1.5 V on charge and discharge, respectively.

Results from electrochemical tests on the Na_2_Fe_2_F_7_ cathode are presented in Figure 5. The voltage profiles for Na_2_Fe_2_F_7_ are shown in Figure 5a through cycle 50. Unless indicated otherwise, Na_2_Fe_2_F_7_ was cycled at a rate of C/20 [full (dis)charge in 20 h assuming the transfer of 2 Na per formula unit] by first charging to 4.3 V against Na^+^/Na and subsequent cycling between 4.3 and 1.5 V. Upon first charge, a capacity of 70 mAh/g is achieved with the following discharge providing a reversible capacity of 125 mAh/g. We note that all capacities reported here are calculated assuming that the entire active material is composed of Na_2_Fe_2_F_7_. This leads to an underestimation of the capacity attributable to the electrochemically active phase since SXRD data collected on the carbon-coated sample suggests the presence of about 10 wt % of crystalline Na_3_FeF_6_ impurity that is largely electrochemically inactive over the potential range probed here. The capacity was computed in this manner as the carbon-coated material likely contains some amount of amorphous electrochemically active domains, which SXRD is unable to detect. While more Na may be able to be intercalated upon discharging the cell to a lower potential, the discharge cutoff voltage was maintained at 1.5 V to prevent the Na_3_FeF_6_ impurity phase from becoming electrochemically active at lower potentials^23^ and interfering with the analysis of the electrochemical behavior of Na_2_Fe_2_F_7_. Beyond the first cycle, the capacity fades steadily (Figure S8a). Differential capacity (d*Q*/d*V*) plots recorded over the first 50 cycles are shown in Figure 5b. During the first few cycles, several broad features are observed on charge and discharge, which gradually evolve into two sharp features centered around 3.3/3.6 and 2.8/3.1 V on discharge/charge. The average discharge voltage (Figure S8b) begins at about 2.8 V but gradually fades to 2.7 V, and the charge–discharge voltage hysteresis increases from 0.5 to 0.75 V after 50 cycles. The rate performance is shown in Figure 5c. A respectable capacity of 85 mAh/g is achieved at a rate of C/5, but the capacity quickly drops off as the rate is increased to 1C, suggesting that Na (de)intercalation from/into the weberite cathode is kinetically limited. Galvanostatic intermittent titration technique (GITT) tests were performed to understand the extent of charge-transfer and Na-ion conduction limitations, which additively contribute to the observed overpotential,^49^ with results shown in Figure 5d. At high and low voltages, large overpotentials (approaching 0.5 V) are observed, while overpotentials are minimal throughout the rest of the electrochemical profile. This suggests that the processes between 2.5 and 4 V, approximately corresponding to Na (de)intercalation and Fe redox between the compositional bounds of Na_2_Fe^2+^Fe^3+^F_7_ and Na_1_Fe^3+^_2_F_7_, are kinetically facile, while Na (de)intercalation and Fe redox beyond these bounds are hindered. Even after a 2 h equilibration period after each current pulse, a significant voltage hysteresis remains throughout the electrochemical profile, suggesting some asymmetry associated with the charge–discharge processes, which will be revisited in the following sections.

Overall, the present Na_2_Fe_2_F_7_ cathode exhibits respectable capacities over the first few cycles but steady capacity fade upon extended cycling. These results differ from prior reports: Dey *et al.* reported a much lower initial reversible capacity of 58 mAh/g for a Na_2_Fe_2_F_7_ cathode synthesized via a topochemical route,^36^ while Park *et al.* obtained a higher initial capacity and higher cycling stability for a cathode prepared using a very similar synthesis route to the one adopted here.^30^ While those discrepancies may stem from a different ratio of weberite variants in their samples, the electrochemical performance is likely also strongly dependent on the specific cathode preparation method used in each study. Such a strong dependence on electrode formulation and processing has been reported in poor-electron conducting materials, such as transition metal fluoride electrodes for sodium-^20,41,50−52^ and lithium-based^17,19,41^ batteries and also lithium iron phosphate.^42,53,54^

To better understand the origin of the capacity fade observed here and the differences in the electrochemical performance reported in the three studies of Na_2_Fe_2_F_7_, *ex situ* characterization of cathode samples and a computational investigation of the dependence of the electrochemical properties of weberite Na_2_Fe_2_F_7_ on the structural variant are presented in the next sections.

### *Ex Situ* Characterization of the Charge–Discharge Mechanism

**Figure 6:**
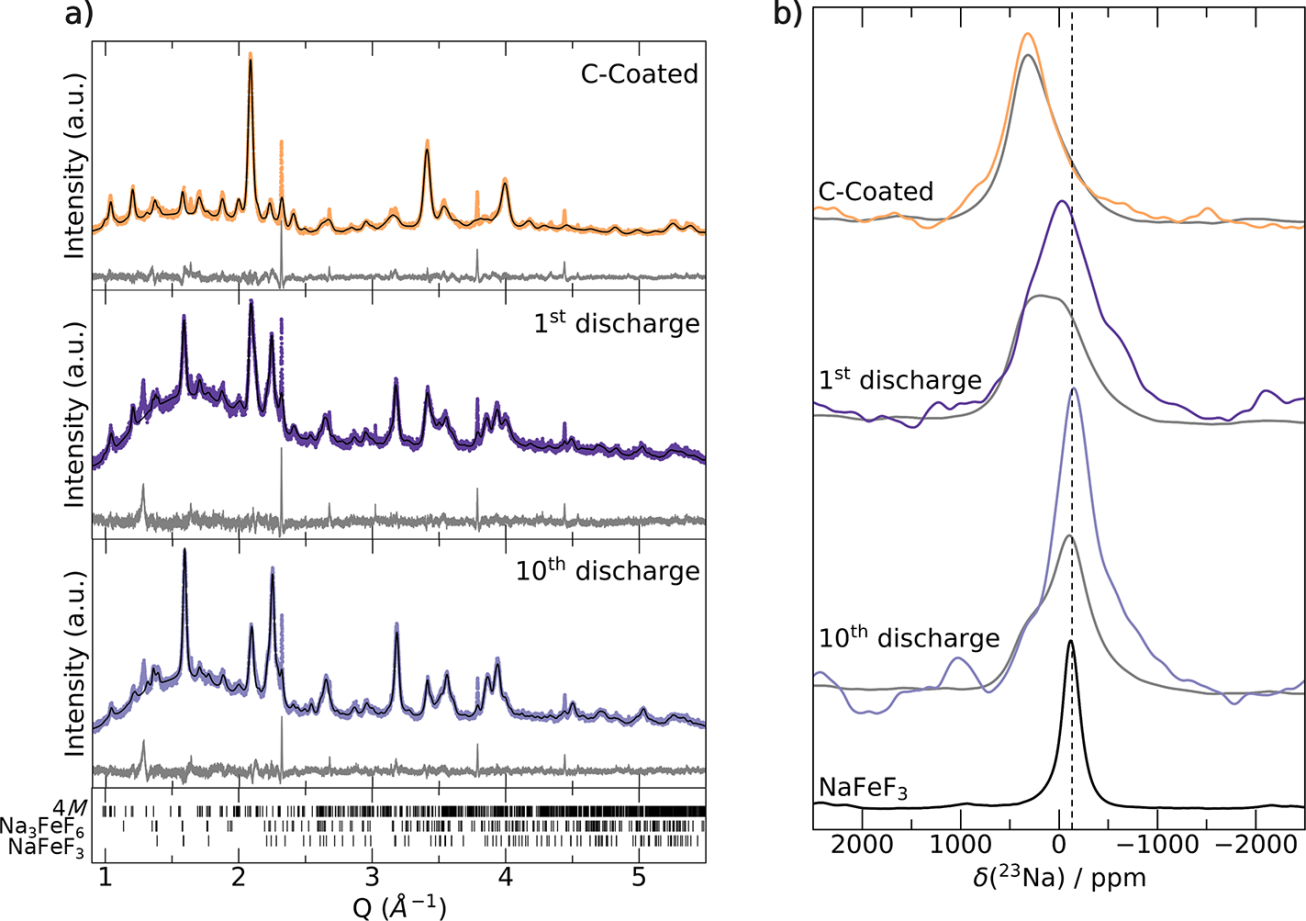
Characterization of *ex situ* Na_*x*_Fe_2_F_7_ cathode samples. (a) SXRD patterns with corresponding Rietveld refinements (black, difference pattern in gray) and (b)^23^Na ss-NMR spin-echo spectra obtained on carbon-coated Na_2_Fe_2_F_7_ (top) and on *ex situ* Na_*x*_Fe_2_F_7_ samples collected after the 1st (middle) and 10th (bottom) discharges. Spectra obtained with a π/2 excitation pulse are shown in gray and those obtained with a π/6 excitation pulse are colored. The bottom ^23^Na ss-NMR spectrum was obtained on NaFeF_3_ using a π/2 pulse. The dashed line indicates the position of the NaFeF_3_ resonance.

The evolution of the d*Q*/d*V* features (Figure 5b) suggests a change in the bulk structure and redox mechanism of the Na_2_Fe_2_F_7_ cathode upon extended cycling. To monitor those structural changes, *ex situ* SXRD and ^23^Na ss-NMR characterization was performed on samples collected at the end of the 1st and 10th discharges, with results shown in Figure 6. Prior to cycling, weberite-type Na_*x*_Fe_2_F_7_ is the main active component of the cathode, with about 10 wt % of Na_3_FeF_6_ present. However, *ex situ* SXRD data indicate a partial phase transformation of the Na_*x*_Fe_2_F_7_ weberite phase during cycling. Rietveld refinements of the SXRD patterns collected after the 1st and 10th cycles (see Rietveld refinement parameters listed in Tables S9 and S10) suggest that the newly formed phase is perovskite-like NaFeF_3_ (*Pnma*), which accounts for 40 wt % of the sample after the 1st discharge and 53 wt % of the sample after the 10th discharge. A constant weight fraction of Na_3_FeF_6_ remains in the sample upon cycling, suggesting that the Na_*y*_FeF_3_ phase evolves at the expense of Na_*x*_Fe_2_F_7_ weberite phases only. Note that the unassigned impurity peaks are still present and do not evolve throughout cycling, suggesting that they correspond to an electrochemically inactive component.

As SXRD is only sensitive to crystalline domains, ^23^Na ss-NMR spectra were also collected on the *ex situ* samples, as shown in Figure 6b. For comparison, NaFeF_3_ was synthesized—its crystal structure, laboratory XRD pattern, and corresponding refinement are shown in Figure S9—and its ^23^Na ss-NMR spectrum is also shown in Figure 6b. The single, broad ^23^Na signal at −118 ppm in the spectrum collected on NaFeF_3_ presumably corresponds to the single, relatively symmetric Na environment in the perovskite-like structure. The NMR spectra collected on Na_*x*_Fe_2_F_7_ cathode samples reveal significant structural changes upon cycling, with the appearance of a signal at −118 ppm consistent with the formation of NaFeF_3_. In line with the SXRD results, the fraction of NaFeF_3_ increases with cycling, as indicated by the gradual growth of the −118 ppm peak. The broader and low-intensity signals in the *ex situ* spectra likely correspond to cubic and bihexagonal pyramidal Na environments in residual Na_*x*_Fe_2_F_7_ domains, which can be differentiated using π/2 and π/6 flip angle spin-echo experiments as discussed previously. Additionally, ^19^F NMR data, shown in Figure S10, indicate a very minor NaF component in the *ex situ* samples. Integration of this signal suggests the presence of <0.05 wt % of NaF in the total sample, and thus, this component likely results from electrolyte decomposition^55,56^ rather than from the phase transformation process.

Thus, our *ex situ* results show that our weberite cathode undergoes a phase transformation to Na_*y*_FeF_3_ (0 ≤ *y* ≤ 1) upon cycling. These findings agree well with the electrochemical results, as Na_*y*_FeF_3_ is electrochemically active with very similar d*Q*/d*V* features and comparable experimental capacities (≈105 mAh/g) as those observed on later cycles as shown in Figure 5b.^57,58^ Furthermore, the several kinks observed in the galvanostatic data during the first discharge process may indicate the beginning of this phase transformation, as shown with select single cycle d*Q*/d*V* plots in Figure S8c–f. As the d*Q*/d*V* curve evolves at least to the 20th cycle, the Na_*x*_Fe_2_F_7_ to Na_*y*_FeF_3_ phase transformation continues up to this point. The electrochemical behavior on later cycles suggests that little to no weberite phase is left after 20 cycles, and the capacity observed is mostly due to Na (de)intercalation from/into Na_*y*_FeF_3_.

Based on the ternary phase diagram presented in Figure 1d, one would anticipate the as-synthesized Na_2_Fe_2_F_7_ weberite cathode to decompose into FeF_2_, NaFeF_4_, and Na_3_FeF_6_. However, the black, dotted tie line in the phase diagram represents the compositional evolution of the weberite at various stages of charge, clearly indicating that on charge and discharge, thermodynamically stable, perovskite-like Na_*y*_FeF_3_ phases (including FeF_3_ and NaFeF_3_) become possible decomposition phases of the metastable weberite cathode, providing a potential explanation for the observed phase transformation. While thermodynamics are important to rationalize phase transformations, kinetics must also be taken into account. Kinetic considerations may provide further insights into the mechanism of the phase transformation. Here, the perovskite-type Na_*y*_FeF_3_ and weberite Na_*x*_Fe_2_F_7_ structures contain similar building blocks, likely facilitating the structural rearrangements. As previously mentioned, weberite Na_*x*_Fe_2_F_7_ contains a 3D network of corner-sharing FeF_6_ octahedra and 1D Fe^2+^F_6_ octahedral chains. Na_*y*_FeF_3_ similarly contains corner-sharing Fe^2+^F_6_ octahedral chains, now connected in 3D, as it forms a perovskite-like structure with Na occupying lattice sites in-between the chains (Figure S9b). Thus, upon Na (de)intercalation from/into the weberite structure, rearrangement of the FeF_6_ octahedra may enable the weberite to perovskite phase transformation. Notably, Fe^3+^ possesses a high spin *d*^5^ electron configuration, hence no octahedral versus tetrahedral site preference, and can easily migrate to nearby sites as has been observed in other Fe-containing cathode materials.^59−62^ Hence, the phase transformation is expected to be both kinetically facile and thermodynamically favored. Additional clues as to the onset and mechanism of the phase transformation come from the electrochemical and *ex situ* SXRD and NMR analysis. First, the sharp and prominent d*Q*/d*V* feature observed at ∼3.1 V during the first discharge process (Figure 5b) is tentatively attributed to the onset of the phase transformation, suggesting that this phase transformation is initiated at a Na content *x* < 2 in the weberite phase. Given that the only two Na-containing phases observed by ^23^Na NMR and SXRD in the discharged samples are the weberite and perovskite phases and ^19^F NMR data further confirms a negligible amount of NaF in these samples, the Na content in the perovskite phase formed on discharge is presumably equal to that in the initial weberite phase. Hence, a possible reaction mechanism is Na_*x*_Fe_2_F_7_ → 2 Na_*x*/2_FeF_3_ + 1/2 F_2(g)_, where F_2(g)_ likely reacts with the electrolyte to form HF. In turn, HF formation during cycling could contribute to the rapid capacity decay. In fact, we have seen evidence of residues on the stainless steel plungers used in the Swagelok cells that are suggestive of corrosion.

The phase transformation observed here appears to be dependent on the specific weberite variant composition and/or the electrode preparation method, as Park *et al.* observed only the weberite phase via *ex situ* XRD after the 1000th cycle.^30^ Regardless, the metastability and polymorphism of weberite compounds, exemplified here for Na_2_Fe_2_F_7_, are important considerations for the development and accurate evaluation of this class of cathode materials.

### Computational Investigation of the Electrochemical Properties of Na_2_Fe_2_F_7_ Weberite Variants

**Figure 7:**
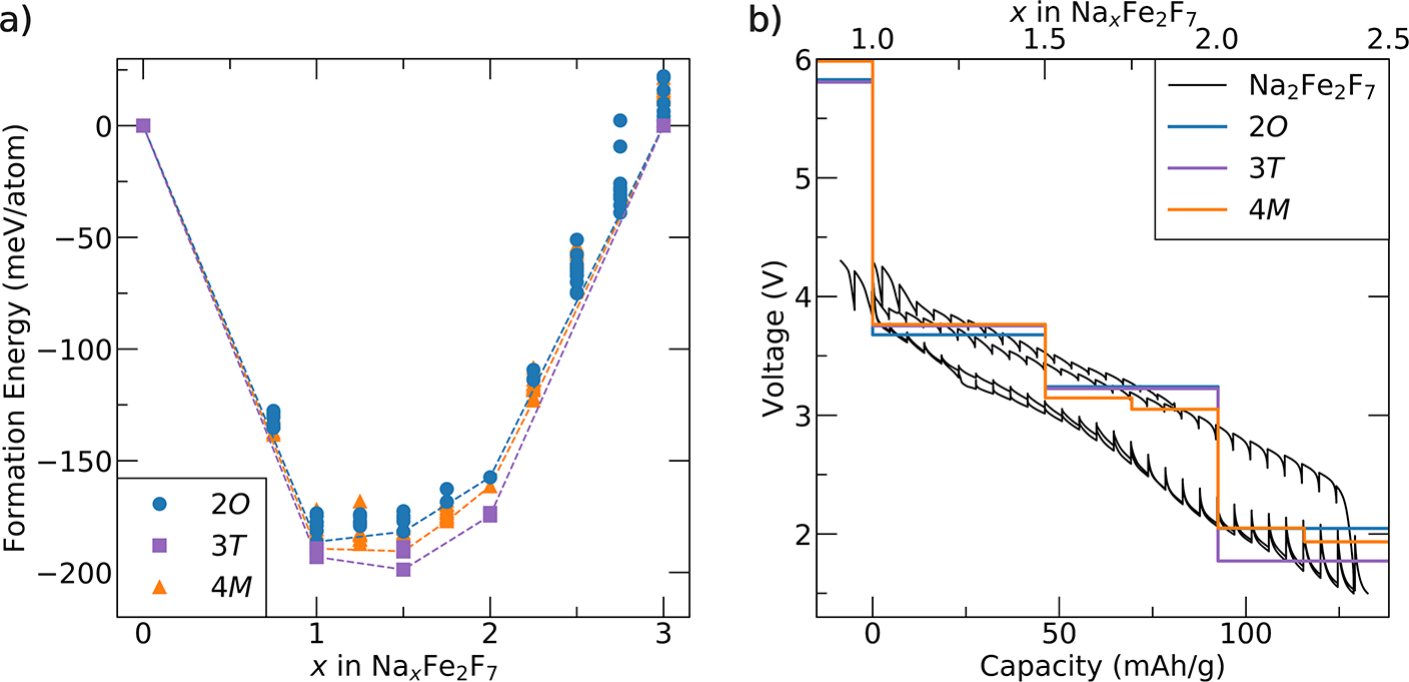
First-principles investigation of the Na (de)intercalation behavior of Na_2_Fe_2_F_7_ polymorphs. (a) Calculated formation energies for 2*O*, 3*T*, and 4*M* Na_*x*_Fe_2_F_7_ structures. The convex hull for each polymorph is shown as a dashed line. (b) Predicted voltage curves for the three weberite polymorphs derived from the 0 K Na_*x*_Fe_2_F_7_ formation energies shown in (a). The GITT data from Figure 5d has been reproduced in black for comparison.

To elucidate the influence of the weberite polymorph identity on the electrochemical performance of Na_2_Fe_2_F_7_, the thermodynamic stability of a series of Na_*x*_Fe_2_F_7_ (0 ≤ *x* ≤ 3) compositions was evaluated from first principles for the 2*O*, 3*T*, and 4*M* variants. The three resulting convex hulls are overlaid in Figure 7a, which include various Na-vacancy orderings at intermediate Na_*x*_Fe_2_F_7_ compositions and polymorph-specific Fe_2_F_7_ and Na_3_Fe_2_F_7_ end-member phases (additional computational details are provided in Note S3). To achieve compositions with *x* > 2, additional Na intercalation sites were identified within the 2*O* and 4*M* Na_2_Fe_2_F_7_ structures using bond valence sum mapping in the SoftBV software program,^63−65^ as these polymorphs do not contain any intrinsic Na vacancies. The coordinates of possible intercalation sites are listed in Table S11. For the 3*T* structure with two half-filled Na sites at *x* = 2, no additional intercalation sites needed to be considered. The three variants have very similar energetics within the 0 ≤ *x* ≤ 3 compositional range (differing by no more than 30 meV/atom), with the 3*T* structure being most stable overall, followed by the 4*M* variant. Notably, the presence of many intermediate Na_*x*_Fe_2_F_7_ compositions on the convex hulls (dashed lines), each with many Na-vacancy orderings within 10–20 meV/atom, suggests that all three weberite variants should undergo a solid solution mechanism leading to a sloped voltage profile (assuming that the weberite structure does not transform during cycling).

From these results, equilibrium voltage curves were generated for the three variants using the Nernst equation given below,^66^ where μ_Na_ is the chemical potential of Na in Na_*x*_Fe_2_F_7_, μ_Na_^o^ is the Na chemical potential in the reference anode, and *e* is the elementary charge
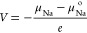


These equilibrium voltage curves are shown in Figure 7b along with the experimental Na_2_Fe_2_F_7_ GITT data reproduced from Figure 5d. The computed curves are only an approximation to the true voltage curves as they are obtained from the 0 K DFT energies of intermediate Na_*x*_Fe_2_F_7_ phases and as such neglect any temperature effects and assume that all intermediate phases have been correctly identified.^66^ Here, the step-like voltage profiles obtained from first principles result from sampling over a small subset of intermediate Na_*x*_Fe_2_F_7_ compositions and Na/vacancy orderings, whereas the smoother, finite-temperature experimental profile is the result of entropic effects that create disorder on the Na sublattice. Overall, the predicted voltage profiles of the three polymorphs are very similar, with average voltages of 3.46, 3.49, and 3.43 V between *x* = 1 and 2 and of 2.75, 2.63, and 2.7 V between *x* = 1 and 3 for the 2*O*, 3*T*, and 4*M* polymorphs. In addition, the predicted profiles align well with experimental results. Below *x* = 1, large overpotentials are observed in the GITT data, which our DFT results suggest is due to the high potentials (>5.75 V) required for further Na extraction, well outside the electrochemical stability window of conventional carbonate electrolytes.^67−69^ The high predicted potentials at the top of charge arise from a rather unstable fully deintercalated structure, which is likely partly due to the difficulty of oxidizing Fe past Fe^3+^.^70,71^ In fact, Fe^4+^ has never been observed in fluoride materials. Large overpotentials are also observed in the GITT data at the end of discharge (*x* > 2), although our calculations suggest that Na should be able to intercalate up to *x* = 3 within the potential range used in our experiments. Thus, Na intercalation past *x* = 2 appears to be kinetically hindered in our experiments, which may in part be due to the large volume expansion (upward of 10%) predicted past *x* = 2 (as shown in Figure S11) or by the phase transformation to Na_*y*_FeF_3_.

Overall, our DFT calculations predict a very similar electrochemical behavior for the three weberite structural variants, suggesting that the different layer stackings do not have a significant influence on the range of stoichiometries accessible via Na (de)intercalation. While we did not investigate the Na-ion conduction behavior within each polymorph, the slight structural variations among 2*O*, 3*T*, and 4*M* Na_*x*_Fe_2_F_7_ likely do not affect Na-ion transport drastically, especially considering that the Na polyhedral connectivity is largely retained in each structural variant and the slow cycling rate used here (C/20). However, a more detailed study of the Na-ion transport properties of the three weberite variants is warranted.

While the origin of the very different electrochemical behaviors reported here and in Park *et al.*’s work^30^ is difficult to ascertain, particularly since we are unable to compare the Na_2_Fe_2_F_7_ polymorph ratios present in the starting cathodes due to the significant broadening of the diffraction patterns, we suspect that the different electrode preparation methods employed in the two studies play a large role, and kinetic limitations in our electrode films encourage a phase transformation to perovskite Na_*y*_FeF_3_ to occur, rather than topotactic Na intercalation. Thus, to prevent transformation of the weberite phase, these kinetic limitations must be overcome, and/or the weberite phase must be stabilized with respect to competing phases.

## Conclusions

This work comprises an in-depth examination of the structure, phase stability, and electrochemical performance of the Na_2_Fe_2_F_7_ weberite cathode, using a combined experimental–computational approach. First-principles calculations reveal that Na_2_Fe_2_F_7_ is metastable and highly prone to polymorphism, as confirmed by Rietveld analysis of the synchrotron XRD data collected on the pristine Na_2_Fe_2_F_7_ sample, indicating a mixture of the orthorhombic (2*O*), trigonal (3*T*), and monoclinic (4*M*) weberite polymorphs. These results are consistent with the multiple Fe environments observed by ^57^Fe Mössbauer spectroscopy and the cubic and bihexagonal pyramidal Na environments identified by ^23^Na solid-state NMR and first-principles calculations of NMR parameters. The Na_2_Fe_2_F_7_ cathode exhibits an initial reversible capacity of 125 mAh/g and a 60% capacity retention after 50 cycles. Contributing to the capacity fade is a transformation of the Na_2_Fe_2_F_7_ weberite phases to the Na_*y*_FeF_3_ perovskite, as revealed via *ex situ* synchrotron XRD and ^23^Na solid-state NMR. A first-principles investigation of the impact of polymorphism on the electrochemical performance reveals that the orthorhombic (2*O*), trigonal (3*T*), and monoclinic (4*M*) Na_2_Fe_2_F_7_ polymorphs should behave similarly, transferring up to 2 Na from Na_1_Fe_2_F_7_ to Na_3_Fe_2_F_7_ at an average voltage of 2.7 V. Thus, from a thermodynamic standpoint, the polymorphic makeup of the Na_2_Fe_2_F_7_ weberite cathode does not have a strong impact on the electrochemical behavior, although future investigations of the Na-ion transport properties of the weberite variants are warranted. We suspect that the poorer electrochemical performance of our Na_2_Fe_2_F_7_ cathode compared to that reported in a previous study by Park *et al.* is largely due to differences in cathode film formulation and preparation methods. In this work, poorer overall kinetics hinder topotactic Na-ion (de)intercalation and favor a phase transformation reaction. Therefore, detailed reports regarding composite electrode preparation protocols and a careful analysis of the structure of weberite compounds are needed to assist the further development of this new class of Na-ion cathodes.

## Methods

### Material Synthesis

Na_2_Fe_2_F_7_ was prepared via a mechanochemical-assisted solid-state route, using a stoichiometric mixture of dried binary fluoride precursors: NaF (Sigma-Aldrich, 99.99%), FeF_2_ (Sigma-Aldrich, 99%), and FeF_3_ (Sigma-Aldrich, 99.5%). All precursors and prepared materials were handled in an argon glovebox or else sealed under argon at all times as fluorides react readily with water to form hydrofluoric acid. The precursors were hand-mixed, and then, 1 g of the powder was sealed in a 50 mL ZrO_2_ ball mill jar with 5 10 mm and 10 5 mm ZrO_2_ balls and ball-milled at 400 rpm for 36 h to ensure homogeneous mixing. The resultant powder was pelletized, annealed at 500 °C for 30 min under an argon flow, and then immediately quenched. To prevent air and water exposure, the quench was performed by using a long alumina tube that was shifted horizontally in the tube furnace following the annealing such that the pellet was no longer within the heating element. Subsequently, the alumina tube surrounding the pellet was flushed with room-temperature nitrogen gas for 10 min. The pellet was then hand-ground, and the resulting powder used for all further characterization.

### Material Characterization

#### X-ray Diffraction

High-resolution synchrotron powder diffraction patterns were collected on Beamline 11-BM at the Advanced Photon Source (APS), Argonne National Laboratory, using an average wavelength of 0.458940 Å. Room-temperature data were collected between 2θ of 0.5 and 50°. Resulting patterns were refined using first the Pawley method, to determine accurate peak shape fitting parameters, and then using the Rietveld method in TOPAS v7.^72^ Crystal structures were depicted using VESTA 3.^73^

#### Scanning Electron Microscopy

SEM images were obtained using a Thermo Fisher Apreo C LoVac SEM instrument with an accelerating voltage of 5 keV and a current of 0.4 nA.

#### ^57^Fe Mössbauer Spectroscopy

Room-temperature ^57^Fe Mössbauer spectroscopy was performed using a SEECo Model W304 resonant gamma-ray spectrometer (activity = 60 mCi ± 10%, ^57^Co/Rh source) equipped with a Janis Research Model SVT-400 cryostat system. The source linewidth was <0.12 mm s^–1^ for the innermost lines of a 25 μm α-Fe foil standard. Isomer shifts were referred to the α-Fe foil at room temperature. All samples consisted of 15–20 mg of the material loaded into a plastic holder in an Ar glovebox, coated with oil, capped, and then measured under a positive flow of N_2_ at 305 K. The data were fit using MossA.^74^

#### Inductively Coupled Plasma Mass Spectrometry

Bulk chemical compositions were determined via ICP (Agilent 5800 ICP-OES). Samples were dissolved in a solution consisting of a 10:1 (v/v) ratio of 70% HNO_3_ (Sigma-Aldrich) and concentrated HCl (Sigma-Aldrich).

#### ^23^Na Solid-State Nuclear Magnetic Resonance Spectroscopy

^23^Na ssNMR data were collected on the Na_2_Fe_2_F_7_ as-prepared and *ex situ* samples using a Bruker Avance 100 MHz (2.35 T) wide-bore NMR spectrometer with Larmor frequencies of 26.48 MHz at room temperature. The data were obtained at 60 kHz magic-angle spinning (MAS) using a 1.3 mm double-resonance HX probe. ^23^Na NMR data were referenced against 1 M aqueous solutions of sodium chloride [NaCl, δ(^23^Na) = 0 ppm], and these samples were also used for pulse calibration. ^23^Na spin-echo spectra were acquired on all samples using π/2−π–π/2 and π/6−π/3−π/6 pulse sequences to selectively excite less quadrupolar environments and equally excite all environments, respectively. The radiofrequency (RF) pulse lengths were 0.125 μs for π/6, 0.25 μs for π/3, 0.375 μs for π/2, and 0.75 μs for π at a power of 62.5 W. A recycle delay between 30 and 80 ms was used with the exact value optimized for each sample to ensure that the full ^23^Na signal was fully relaxed between pulses.

#### ^19^F Solid-State Nuclear Magnetic Resonance Spectroscopy

^19^F ssNMR data were collected on the Na_2_Fe_2_F_7_*ex situ* samples using a Bruker Avance 100 MHz (2.35 T) wide-bore NMR spectrometer with Larmor frequencies of 94.08 MHz at room temperature. The data were obtained at 60 kHz MAS using a 1.3 mm double-resonance HX probe. ^19^F NMR data were referenced against 1 M aqueous solutions of sodium fluoride [NaF, δ(^19^F) = −118.14 ppm], and these samples were also used for pulse calibration. The RF pulse lengths were 0.35 μs for π/2 and 0.7 μs for π. A recycle delay of 60 s was used for each sample to ensure that the full ^19^F signal was fully relaxed between pulses.

#### Electrochemical Characterization

The as-synthesized Na_2_Fe_2_F_7_ materials were carbon-coated prior to electrochemical testing to form an electronically conductive carbon nanocomposite. The pristine materials were combined with carbon black (Super C65, MTI Corporation) in a 7:2 ratio along with three 10 mm and three 5 mm ZrO_2_ balls in a 50 mL ZrO_2_ ball mill jar and ball-milled at 300 rpm for 24 h. The resultant carbon-coated materials were hand-ground for 30 min with 10 wt % polytetrafluoroethylene (Sigma-Aldrich), rolled into free-standing film electrodes, and punched into 6 mm disks with a loading density of 10 mg cm^–2^. All electrochemical testing occurred in Swagelok-type cells against Na metal (Sigma) with a glass fiber separator (Whatman GF/D) using 200 μL of an in-house-prepared electrolyte (<20 ppm water content) of 1 M NaPF_6_ (Strem Chemicals, 99%) in propylene carbonate (Sigma, 99.7%) with 2 vol % fluoroethylene carbonate (Sigma-Aldrich, ≥99%).

### Computational Details

#### Phase Stability and Energetics

DFT calculations were performed using the Vienna *ab initio* Simulation Package (VASP).^75−78^ All VASP calculations used projector augmented wave pseudopotentials (Na pv, Fe, and F),^79,80^ a plane-wave energy cutoff of 520 eV, and the Perdew–Burke–Ernzerhof generalized-gradient approximation^81^ functional with the Hubbard *U* correction.^82^ A *U* value of 4 eV was used for Fe based on previous reports that have shown it to be broadly reliable for ionic solids.^83,84^

All calculations were performed on a 1 × 1 × 1 cell of the Na_*x*_Fe_2_F_7_ 2*O*, 3*T*, and 4*M* structures. For structures containing partial occupation of Na sites, symmetrically unique Na-vacancy orderings were enumerated and ranked according to their Ewald sum energy as implemented in Pymatgen^85^ and only the three lowest-energy structures considered. The structures were fully optimized (atomic positions and cell parameters), and the theoretical lattice parameters are compared to experimental values^30,36,37^ in Table S8. To obtain accurate final energies, all relaxations were followed by a final static calculation. The convergence criteria were set as 10^–5^ eV for total energy and 0.01 eV/Å for the interatomic forces. Gaussian smearing with a width of 0.05 eV was used. All calculations were spin-polarized with ferromagnetic ordering assumed. For the 2*O* and 3*T* structures, the Brillouin zone was sampled with a 4 × 3 × 4 and 6 × 6 × 3 k-point grid, while the 4*M* structure used a reciprocal space discretization of 25 Å^–1^.

#### Calculation of NMR Parameters

Spin-unrestricted hybrid DFT/HF calculations were performed using the CRYSTAL17 all-electron linear combination of the atomic orbital code^86^ to determine ^23^Na NMR parameters for the Na_2_Fe_2_F_7_ 2*O*, 3*T*, and 4*M* structures optimized in VASP, as described above, which were further relaxed in CRYSTAL. Two spin-polarized exchange–correlation functionals based upon the B3LYP form^87−90^ and with Fock exchange weights of *F*_0_ = 20% (B3LYP or H20) and 35% (H35) were chosen for their good performance regarding the prediction of the electronic structure and band gaps of transition metal compounds (B3LYP or H20)^91,92^ and for their accurate description of the magnetic properties of related compounds (H35).^93−95^ All-electron atom-centered basis sets comprising fixed contractions of Gaussian primitive functions were employed throughout. Two types of basis sets were used: a smaller basis set (BS-I) was employed for structural optimizations, and a larger basis set (BS-II) was used for computing ^23^Na NMR parameters which require an accurate description of the occupation of core-like electronic states. For BS-I, individual atomic sets are of the form (15s7p)/[1s3sp] for Na, (20s12p5d)/[1s4sp2d] for Fe, and (10s6p1d)/[4s3p1d] for F, where the values in parentheses denote the number of Gaussian primitives and the values in square brackets the contraction scheme. All BS-I sets were obtained from the CRYSTAL online repository and were unmodified from their previous use in a broad range of compounds.^96^ For BS-II, a flexible and extended TSDP-derived (11s7p)/[7s3p] set for Na, an Ahlrichs DZP-derived^97^ (13s9p5d)/[7s5p3d] set for Fe, and a modified IGLO-III and (10s6p2d)/[6s5p2d] set for F were used.

NMR parameters were computed on the fully optimized (atomic positions and cell parameters) Na_2_Fe_2_F_7_ structures. All first-principles structural optimizations were carried out in the ferromagnetic state on 1 × 1 × 1 cells (containing 44, 66, and 176 atoms for 2*O*, 3*T*, and 4*M* Na_2_Fe_2_F_7_, respectively), after removal of all symmetry constraints and using the H20 and H35 hybrid functionals. Structural optimizations were pursued using the quasi-Newton algorithm with root-mean-square (RMS) convergence tolerances of 10^–7^, 0.0003, and 0.0012 a.u. for total energy, RMS force, and RMS displacement, respectively. Tolerances for maximum force and displacement components were set to 1.5 times the respective RMS values. Sufficient convergence in total energies and spin densities was obtained by application of integral series truncation thresholds of 10^–7^, 10^–7^, 10^–7^, 10^–7^, and 10^–14^ for Coulomb overlap and penetration, exchange overlap, and g- and n-series exchange penetration, respectively, as defined in the CRYSTAL17 documentation.^96^ The final total energies and spin and charge distributions were obtained in the absence of any spin and eigenvalue constraints. NMR parameters were obtained on ferromagnetically aligned 2 × 1 × 2, 1 × 1 × 1, and 1 × 1 × 1 cells for 2*O*, 3*T*, and 4*M* Na_2_Fe_2_F_7_, respectively, and on cells where one Fe spin was flipped using BS-II sets and a method described by Middlemiss *et al.*(98) Anisotropic Monkhorst–Pack reciprocal space meshes^99^ with shrinking factors of 9 6 9 for 2*O*, 9 9 4 for 3*T*, and 9 6 3 for 4*M* were used throughout.

## Supporting Information Available

Crystal structure diagrams, simulated diffraction patterns, synthesis and electrochemical optimization results, Rietveld refinement results, Mossbauer fit parameters, ICP results, NMR calculation details and results, computational details and results, and additional NMR and electrochemical data (PDF)

## References

[ref1] Clément R. J.; Bruce P. G.; Grey C. P. Review-Manganese-Based P2-Type Transition Metal Oxides as Sodium-Ion Battery Cathode Materials; Journal of the Electrochemical Society; Electrochemical Society Inc, 2015; pp A2589–A2604.

[ref2] Rudola A.; Rennie A. J. R.; Heap R.; Meysami S. S.; Lowbridge A.; Mazzali F.; Sayers R.; Wright C. J.; Barker J. Commercialisation of High Energy Density Sodium-Ion Batteries: Faradion’s Journey and Outlook. J. Mater. Chem. A 2021, 9, 8279–8302. 10.1039/d1ta00376c.

[ref3] Yabuuchi N.; Kajiyama M.; Iwatate J.; Nishikawa H.; Hitomi S.; Okuyama R.; Usui R.; Yamada Y.; Komaba S. P2-Type Nax[Fe1/2Mn1/2]O2 Made from Earth-Abundant Elements for Rechargeable Na Batteries. Nat. Mater. 2012, 11, 512–517. 10.1038/nmat3309. 22543301

[ref4] Goodenough J. B.; Hong H. Y. P.; Kafalas J. A. Fast Na+-Ion Transport in Skeleton Structures. Mater. Res. Bull. 1976, 11, 203–220. 10.1016/0025-5408(76)90077-5.

[ref5] Wu V. C.; Giovine R.; Foley E. E.; Finzel J.; Balasubramanian M.; Sebti E.; Mozur E. M.; Kwon A. H.; Clément R. J. Unlocking New Redox Activity in Alluaudite Cathodes through Compositional Design. Chem. Mater. 2022, 34, 4088–4103. 10.1021/acs.chemmater.2c00324.

[ref6] Masquelier C.; Croguennec L. Polyanionic (Phosphates, Silicates, Sulfates) Frameworks as Electrode Materials for Rechargeable Li (or Na) Batteries. Chem. Rev. 2013, 113, 6552–6591. 10.1021/cr3001862. 23742145

[ref7] Lu Y.; Wang L.; Cheng J.; Goodenough J. B. Prussian Blue: A New Framework of Electrode Materials for Sodium Batteries. Chem. Commun. 2012, 48, 6544–6546. 10.1039/c2cc31777j. 22622269

[ref8] Lim C. Q. X.; Tan Z. K. Prussian White with Near-Maximum Specific Capacity in Sodium-Ion Batteries. ACS Appl. Energy Mater. 2021, 4, 6214–6220. 10.1021/acsaem.1c00987.

[ref9] Tapia-Ruiz N.; Armstrong A. R.; Alptekin H.; Amores M. A.; Au H.; Barker J.; Boston R.; Brant W. R.; Brittain J. M.; Chen Y.; Chhowalla M.; Choi Y. S.; Costa S. I. R.; Crespo Ribadeneyra M.; Cussen S. A.; Cussen E. J.; David W. I. F.; Desai A. V.; Dickson S. A. M.; Eweka E. I.; Forero-Saboya J. D.; Grey C. P.; Griffin J. M.; Gross P.; Hua X.; Irvine J. T. S.; Johansson P.; Jones M. O.; Karlsmo M.; Kendrick E.; Kim E.; Kolosov O. V.; Li Z.; Mertens S. F. L.; Mogensen R.; Monconduit L.; Morris R. E.; Naylor A. J.; Nikman S.; O’Keefe C. A.; Ould D. M. C.; Palgrave R. G.; Poizot P.; Ponrouch A.; Renault S.; Reynolds E. M.; Rudola A.; Sayers R.; Scanlon D. O.; Sen S.; Seymour V. R.; Silván B.; Sougrati M. T.; Stievano L.; Stone G. S.; Thomas C. I.; Titirici M. M.; Tong J.; Wood T. J.; Wright D. S.; Younesi R. 2021 roadmap for sodium-ion batteries. J. Phys. Energy 2021, 3, 031503 10.1088/2515-7655/AC01EF.

[ref10] Lee J.; Papp J. K.; Clément R. J.; Sallis S.; Kwon D.-H.; Shi T.; Yang W.; McCloskey B. D.; Ceder G. Mitigating Oxygen Loss to Improve the Cycling Performance of High Capacity Cation-Disordered Cathode Materials. Nat. Commun. 2017, 8, 981 10.1038/s41467-017-01115-0. 29042560 PMC5645360

[ref11] Clément R. J.; Kitchaev D.; Lee J.; Ceder G. Short-Range Order and Unusual Modes of Nickel Redox in a Fluorine-Substituted Disordered Rocksalt Oxide Lithium-Ion Cathode. Chem. Mater. 2018, 30, 6945–6956. 10.1021/acs.chemmater.8b03794.

[ref12] Huang H.; Faulkner T.; Barker J.; Saidi M. Y. Lithium Metal Phosphates, Power and Automotive Applications. J. Power Sources 2009, 189, 748–751. 10.1016/J.JPOWSOUR.2008.08.024.

[ref13] Recham N.; Chotard J.-N.; Dupont L.; Delacourt C.; Walker W.; Armand M.; Tarascon J.-M. A 3.6 V Lithium-Based Fluorosulphate Insertion Positive Electrode for Lithium-Ion Batteries. Nat. Mater. 2010, 9, 68–74. 10.1038/nmat2590. 19946280

[ref14] Nishijima M.; Gocheva I. D.; Okada S.; Doi T.; Yamaki J.; Nishida T. Cathode Properties of Metal Trifluorides in Li and Na Secondary Batteries. J. Power Sources 2009, 190, 558–562. 10.1016/j.jpowsour.2009.01.051.

[ref15] Dimov N.; Nishimura A.; Chihara K.; Kitajou A.; Gocheva I. D.; Okada S. Transition Metal NaMF3 Compounds as Model Systems for Studying the Feasibility of Ternary Li-M-F and Na-M-F Single Phases as Cathodes for Lithium-Ion and Sodium-Ion Batteries. Electrochim. Acta 2013, 110, 214–220. 10.1016/j.electacta.2013.05.103.

[ref16] Amatucci G. G.; Pereira N. Fluoride Based Electrode Materials for Advanced Energy Storage Devices. J. Fluorine Chem. 2007, 128, 243–262. 10.1016/j.jfluchem.2006.11.016.

[ref17] Lemoine K.; Hémon-Ribaud A.; Leblanc M.; Lhoste J.; Tarascon J. M.; Maisonneuve V. Fluorinated Materials as Positive Electrodes for Li- and Na-Ion Batteries. Chem. Rev. 2022, 122, 14405–14439. 10.1021/acs.chemrev.2c00247. 35969894

[ref18] Li H.; Zhou H. Enhancing the Performances of Li-Ion Batteries by Carbon-Coating: Present and Future. Chem. Commun. 2012, 48, 1201–1217. 10.1039/C1CC14764A. 22125795

[ref19] Badway F.; Pereira N.; Cosandey F.; Amatucci G. G. Carbon-Metal Fluoride Nanocomposites: Structure and Electrochemistry of FeF3:C. J. Electrochem. Soc. 2003, 150, A1209 10.1149/1.1596162.

[ref20] Li C.; Gu L.; Tong J.; Maier J. Carbon Nanotube Wiring of Electrodes for High-Rate Lithium Batteries Using an Imidazolium-Based Ionic Liquid Precursor as Dispersant and Binder: A Case Study on Iron Fluoride Nanoparticles. ACS Nano 2011, 5, 2930–2938. 10.1021/nn1035608. 21375268

[ref21] Ponrouch A.; Cabana J.; Dugas R.; Slack J. L.; Palacín M. R. Electroanalytical Study of the Viability of Conversion Reactions as Energy Storage Mechanisms. RSC Adv. 2014, 4, 35988–35996. 10.1039/C4RA05189K.

[ref22] Wang F.; Robert R.; Chernova N. A.; Pereira N.; Omenya F.; Badway F.; Hua X.; Ruotolo M.; Zhang R.; Wu L.; Volkov V.; Su D.; Key B.; Whittingham M. S.; Grey C. P.; Amatucci G. G.; Zhu Y.; Graetz J. Conversion Reaction Mechanisms in Lithium Ion Batteries: Study of the Binary Metal Fluoride Electrodes. J. Am. Chem. Soc. 2011, 133, 18828–18836. 10.1021/ja206268a. 21894971

[ref23] Foley E. E.; Wong A.; Vincent R. C.; Manche A.; Zaveri A.; Gonzalez-Correa E.; Ménard G.; Clément R. J. Probing Reaction Processes and Reversibility in Earth-Abundant Na3FeF6 for Na-Ion Batteries. Phys. Chem. Chem. Phys. 2021, 23, 20052–20064. 10.1039/d1cp02763h. 34231590

[ref24] He K.; Zhou Y.; Gao P.; Wang L.; Pereira N.; Amatucci G. G.; Nam K. W.; Yang X. Q.; Zhu Y.; Wang F.; Su D. Sodiation via Heterogeneous Disproportionation in FeF2 Electrodes for Sodium-Ion Batteries. ACS Nano 2014, 8, 7251–7259. 10.1021/nn502284y. 24911154

[ref25] Ni D.; Fang L.; Sun W.; Shi B.; Chen X.; Li H.; Wang Z.; Sun K. FeF2@MHCS Cathodes with High Capacity and Fast Sodium Storage Based on Nanostructure Construction. ACS Appl. Energy Mater. 2020, 3, 10340–10348. 10.1021/acsaem.0c00876.

[ref26] Zheng Y.; Hwang J.; Matsumoto K.; Hagiwara R. Electrochemical and Structural Behavior of Trirutile-Derived FeF3during Sodiation and Desodiation. ACS Appl. Energy Mater. 2022, 5, 3137–3145. 10.1021/acsaem.1c03756.

[ref27] Arai H.; Okada S.; Sakurai Y.; Yamaki J. I. Cathode Performance and Voltage Estimation of Metal Trihalides. J. Power Sources 1997, 68, 716–719. 10.1016/S0378-7753(96)02580-3.

[ref28] Kitajou A.; Ishado Y.; Yamashita T.; Momida H.; Oguchi T.; Okada S. Cathode Properties of Perovskite-Type NaMF3 (M = Fe, Mn, and Co) Prepared by Mechanical Ball Milling for Sodium-Ion Battery. Electrochim. Acta 2017, 245, 424–429. 10.1016/J.ELECTACTA.2017.05.153.

[ref29] Gocheva I. D.; Nishijima M.; Doi T.; Okada S.; Yamaki J.; Nishida T. Mechanochemical Synthesis of NaMF3 (M = Fe, Mn, Ni) and Their Electrochemical Properties as Positive Electrode Materials for Sodium Batteries. J. Power Sources 2009, 187, 247–252. 10.1016/j.jpowsour.2008.10.110.

[ref30] Park H.; Lee Y.; Cho M.-K.; Kang J.; Ko W.; Jung Y. H.; Jeon T.-Y.; Hong J.; Kim H.; Myung S.-T.; Kim J. Na2Fe2F7: A Fluoride-Based Cathode for High Power and Long Life Na-Ion Batteries. Energy Environ. Sci. 2021, 14, 1469–1479. 10.1039/d0ee02803g.

[ref31] Kang J.; Ahn J.; Park H.; Ko W.; Lee Y.; Lee S.; Lee S.; Jung S.-K.; Kim J.; Kang J.; Ahn J.; Park H.; Ko W.; Lee Y.; Lee S.; Kim J.; Jung S.-K. Highly Stable Fe2+/Ti3+-Based Fluoride Cathode Enabling Low-Cost and High-Performance Na-Ion Batteries. Adv. Funct. Mater. 2022, 32, 2201816 10.1002/ADFM.202201816.

[ref32] Liao J.; Han J.; Xu J.; Du Y.; Sun Y.; Duan L.; Zhou X. Scalable Synthesis of Na2MVF7 (M = Mn, Fe, and Co) as High- Performance Cathode Materials for Sodium-Ion Batteries. Chem. Commun. 2021, 57, 11497–11500. 10.1039/D1CC04449D. 34651621

[ref33] Tressaud A.; Dance J. M.; Portier J.; Hagenmuller P. Interactions Magnetiques Dans Les Fluorures de Type Weberite. Mat. Res. Bull 1974, 9, 1219–1226. 10.1016/0025-5408(74)90040-3.

[ref34] Cosier R.; Wise A.; Tressaud A.; Grannec J.; Olazcuaga R.; Portier J. Sur de Nouveaux Composés Fluorés Ferrimagnétiques à Structure Wébérite. C. R. Hebd. Seances Acad. Sci. C 1970, 271, 142–145.

[ref35] Euchner H.; Clemens O.; Reddy M. A. Unlocking the Potential of Weberite-Type Metal Fluorides in Electrochemical Energy Storage. npj Comput. Mater. 2019, 5, 31 10.1038/s41524-019-0166-3.

[ref36] Dey U. K.; Barman N.; Ghosh S.; Sarkar S.; Peter S. C.; Senguttuvan P. Topochemical Bottom-Up Synthesis of 2D- and 3D-Sodium Iron Fluoride Frameworks. Chem. Mater. 2019, 31, 295–299. 10.1021/acs.chemmater.8b04010.

[ref37] Yakubovich O.; Urusov V.; Massa W.; Frenzen G.; Babel D. Structure of Na2Fe2F7 and Structural Relations in the Family of Weberites Na2MIIMIIIF7. Z. Anorg. Allg. Chem. 1993, 619, 1909–1919. 10.1002/zaac.19936191115.

[ref38] Grey I. E.; Mumme W. G.; Ness T. J.; Roth R. S.; Smith K. L. Structural Relations between Weberite and Zirconolite Polytypes—Refinements of Doped 3T and 4M Ca2Ta2O7 and 3T CaZrTi2O7. J. Solid State Chem. 2003, 174, 285–295. 10.1016/S0022-4596(03)00222-6.

[ref39] Peschel B.; Molinier M.; Babel D. Kristallstrukturbestimmungen an Vier Monoklinen Weberiten Na2MIIMIIIF7 (MII = Fe, Co; MIII = V, Cr). Z. Anorg. Allg. Chem. 1995, 621, 1573–1581. 10.1002/zaac.19956210923.

[ref40] Dahlke P.; Peschel B.; Babel D. Über Röntgenographische Einkristalluntersuchungen an Na2FeAlF7, Na2MIIGaF7 (MII = Ni, Zn) Und Na2ZnFeF7 Und Die Strukturchemie Der Weberite. Z. Anorg. Allg. Chem. 1998, 624, 1003–1010. 10.1002/(sici)1521-3749(199806)624:6<1003::aid-zaac1003>3.0.co;2-c.

[ref41] Badway F.; Cosandey F.; Pereira N.; Amatucci G. G. Carbon Metal Fluoride Nanocomposites: High-Capacity Reversible Metal Fluoride Conversion Materials as Rechargeable Positive Electrodes for Li Batteries. J. Electrochem. Soc. 2003, 150, A1318 10.1149/1.1602454.

[ref42] Wang J.; Sun X. Understanding and Recent Development of Carbon Coating on LiFePO 4 Cathode Materials for Lithium-Ion Batteries. Energy Environ. Sci. 2012, 5, 5163–5185. 10.1039/C1EE01263K.

[ref43] Stephens P. W. Phenomenological Model of Anisotropic Peak Broadening in Powder Diffraction. J. Appl. Crystallogr. 1999, 32, 281–289. 10.1107/S0021889898006001.

[ref44] Laligant Y.; Calage Y.; Heger G.; Pannetier J.; Ferey G. Ordered Magnetic Frustration. VII. Na2NiFeF7: Reexamination of Its Crystal Structure in the True Space Group after Corrections from Renninger Effect and Refinement of Its Frustrated Magnetic Structure at 4.2 and 55 K. J. Solid State Chem. 1989, 78, 66–77. 10.1016/0022-4596(89)90128-X.

[ref45] Pebler J.; Schmidt K.; Babel D.; Verscharen W. Mössbauer-Spektroskopische Untersuchungen an Na2MnFeF7/Mössbauer Spectroscopic Investigation of Na2MnFeF7. Z. Naturforsch. 1977, 32, 369–372. 10.1515/znb-1977-0403.

[ref46] Pankhurst Q. A.; Johnson C. E.; Wanklyn B. M. A Mössbauer Study of Paramagnetic Na2MgFeF7. J. Magn. Magn. Mater. 1991, 97, 126–130. 10.1016/0304-8853(91)90170-F.

[ref47] Cai L.; Nino J. C. Complex Ceramic Structures. I. Weberites. Acta Crystallogr. Sect. B Struct. Sci. 2009, 65, 269–290. 10.1107/S0108768109011355. 19461137

[ref48] Wasylishen R. E.; Ashbrook S. E.; Wimperis S. NMR of Quadrupolar Nuclei in Solid Materials; Wiley: Chichester, 2012.

[ref49] Bard A. J.; Faulkner L. R. Electrochemical Methods; Wiley: New York, 2001.

[ref50] Brennhagen A.; Cavallo C.; Wragg D. S.; Vajeeston P.; Sjåstad A. O.; Koposov A. Y.; Fjellvåg H. Operando XRD Studies on Bi2MoO6as Anode Material for Na-Ion Batteries. Nanotechnology 2022, 33, 185402 10.1088/1361-6528/ac4eb5. 35078157

[ref51] Yabuuchi N.; Kubota K.; Aoki Y.; Komaba S. Understanding Particle-Size-Dependent Electrochemical Properties of Li2MnO3-Based Positive Electrode Materials for Rechargeable Lithium Batteries. J. Phys. Chem. C 2016, 120, 875–885. 10.1021/acs.jpcc.5b10517.

[ref52] Jiang C.; Wei M.; Qi Z.; Kudo T.; Honma I.; Zhou H. Particle Size Dependence of the Lithium Storage Capability and High Rate Performance of Nanocrystalline Anatase TiO2 Electrode. J. Power Sources 2007, 166, 239–243. 10.1016/J.JPOWSOUR.2007.01.004.

[ref53] Belharouak I.; Johnson C.; Amine K. Synthesis and Electrochemical Analysis of Vapor-Deposited Carbon-Coated LiFePO4. Electrochem. Commun. 2005, 7, 983–988. 10.1016/j.elecom.2005.06.019.

[ref54] Lepage D.; Sobh F.; Kuss C.; Liang G.; Schougaard S. B. Delithiation Kinetics Study of Carbon Coated and Carbon Free LiFePO4. J. Power Sources 2014, 256, 61–65. 10.1016/J.JPOWSOUR.2013.12.054.

[ref55] Komaba S.; Ishikawa T.; Yabuuchi N.; Murata W.; Ito A.; Ohsawa Y. Fluorinated Ethylene Carbonate as Electrolyte Additive for Rechargeable Na Batteries. ACS Appl. Mater. Interfaces 2011, 3, 4165–4168. 10.1021/AM200973K/SUPPL_FILE/AM200973K_SI_001.PDF. 22026720

[ref56] Chen L.; Kishore B.; Walker M.; Dancer C. E. J.; Kendrick E. Nanozeolite ZSM-5 Electrolyte Additive for Long Life Sodium-Ion Batteries. Chem. Commun. 2020, 56, 11609–11612. 10.1039/D0CC03976D. 32869777

[ref57] Martin A.; Doublet M.-L.; Kemnitz E.; Pinna N.; Martin A.; Kemnitz E.; Pinna N.; Doublet M. Reversible Sodium and Lithium Insertion in Iron Fluoride Perovskites. Adv. Funct. Mater. 2018, 28, 1802057 10.1002/ADFM.201802057.

[ref58] Zheng Y.; Jitto S.; Hwang J.; Matsumoto K.; Hagiwara R. Multiphase Transformation of NaFeF3 During Desodiation and Sodiation. ACS Appl. Energy Mater. 2022, 5, 14361–14371. 10.1021/ACSAEM.2C02904.

[ref59] Lim S. G.; Kwon M. S.; Kim T.; Kim H.; Lee S.; Lim J.; Kim H.; Lee K. T. Correlation between the Cation Disorders of Fe3+and Li+in P3-Type Na0.67[Li0.1(Fe0.5Mn0.5)0.9]O2for Sodium Ion Batteries. ACS Appl. Mater. Interfaces 2022, 14, 33120–33129. 10.1021/acsami.2c05784. 35830246

[ref60] Yabuuchi N.; Yoshida H.; Komaba S. Crystal Structures and Electrode Performance of Alpha-NaFeO 2 for Rechargeable Sodium Batteries. Electrochemistry 2012, 80, 716–719. 10.5796/electrochemistry.80.716.

[ref61] Li Y.; Gao Y.; Wang X.; Shen X.; Kong Q.; Yu R.; Lu G.; Wang Z.; Chen L. Iron Migration and Oxygen Oxidation during Sodium Extraction from NaFeO2. Nano Energy 2018, 47, 519–526. 10.1016/J.NANOEN.2018.03.007.

[ref62] Ye T.; Barpanda P.; Nishimura S. I.; Furuta N.; Chung S. C.; Yamada A. General Observation of Fe3+/Fe2+ Redox Couple Close to 4 v in Partially Substituted Li2FeP2O7 Pyrophosphate Solid-Solution Cathodes. Chem. Mater. 2013, 25, 3623–3629. 10.1021/CM401547Z/SUPPL_FILE/CM401547Z_SI_001.PDF.

[ref63] Chen H.; Adams S. Bond Softness Sensitive Bond-Valence Parameters for Crystal Structure Plausibility Tests. IUCrJ 2017, 4, 614–625. 10.1107/S2052252517010211/YC5011SUP1.PDF. PMC5619853 28989717

[ref64] Chen H.; Wong L. L.; Adams S. SoftBV – a Software Tool for Screening the Materials Genome of Inorganic Fast Ion Conductors. Acta Crystallogr. Sect. B Struct. Sci. 2019, 75, 18–33. 10.1107/S2052520618015718. 32830774

[ref65] Wong L. L.; Phuah K. C.; Dai R.; Chen H.; Chew W. S.; Adams S. Bond Valence Pathway Analyzer-An Automatic Rapid Screening Tool for Fast Ion Conductors within SoftBV. Chem. Mater. 2021, 33, 625–641. 10.1021/acs.chemmater.0c03893.

[ref66] Van Der Ven A.; Deng Z.; Banerjee S.; Ong S. P. Rechargeable Alkali-Ion Battery Materials: Theory and Computation. Chem. Rev. 2020, 120, 6977–7019. 10.1021/acs.chemrev.9b00601. 32022553

[ref67] Yan G.; Alves-Dalla-Corte D.; Yin W.; Madern N.; Gachot G.; Tarascon J.-M. Assessment of the Electrochemical Stability of Carbonate-Based Electrolytes in Na-Ion Batteries. J. Electrochem. Soc. 2018, 165, A1222–A1230. 10.1149/2.0311807jes.

[ref68] Ponrouch A.; Marchante E.; Courty M.; Tarascon J.-M. M.; Palacín M. R. In search of an optimized electrolyte for Na-ion batteries. Energy Environ. Sci. 2012, 5, 8572 10.1039/c2ee22258b.

[ref69] Liang H. J.; Gu Z. Y.; Zhao X. X.; Guo J. Z.; Yang J. L.; Li W. H.; Li B.; Liu Z. M.; Li W. L.; Wu X. L. Ether-Based Electrolyte Chemistry Towards High-Voltage and Long-Life Na-Ion Full Batteries. Angew. Chem., Int. Ed. 2021, 60, 26837–26846. 10.1002/ANIE.202112550. 34636126

[ref70] Li B.; Sougrati M. T.; Rousse G.; Morozov A. V.; Dedryvère R.; Iadecola A.; Senyshyn A.; Zhang L.; Abakumov A. M.; Doublet M. L.; Tarascon J. M. Correlating Ligand-to-Metal Charge Transfer with Voltage Hysteresis in a Li-Rich Rock-Salt Compound Exhibiting Anionic Redox. Nat. Chem. 2021, 13, 1070–1080. 10.1038/s41557-021-00775-2. 34531571

[ref71] Fehse M.; Bessas D.; Mahmoud A.; Diatta A.; Hermann R. P.; Stievano L.; Sougrati M. T. The Fe4+/3+ Redox Mechanism in NaFeO2: A Simultaneous Operando Nuclear Resonance and X-Ray Scattering Study. Batter. Supercaps 2020, 3, 1341–1349. 10.1002/BATT.202000157.

[ref72] Coelho A. A. TOPAS and TOPAS-Academic: An Optimization Program Integrating Computer Algebra and Crystallographic Objects Written in C++. J. Appl. Crystallogr. 2018, 51, 210–218. 10.1107/S1600576718000183.

[ref73] Momma K.; Izumi F. VESTA 3 for Three-Dimensional Visualization of Crystal, Volumetric and Morphology Data. J. Appl. Crystallogr. 2011, 44, 1272–1276. 10.1107/S0021889811038970.

[ref74] Prescher C.; McCammon C.; Dubrovinsky L. MossA: A Program for Analyzing Energy-Domain Mössbauer Spectra from Conventional and Synchrotron Sources. J. Appl. Crystallogr. 2012, 45, 329–331. 10.1107/S0021889812004979.

[ref75] Kresse G.; Hafner J. Ab Initio Molecular Dynamics for Liquid Metals. Phys. Rev. B: Condens. Matter Mater. Phys. 1993, 47, 558–561. 10.1103/PhysRevB.47.558. 10004490

[ref76] Kresse G.; Hafner J. Ab Initio Molecular-Dynamics Simulation of the Liquid-Metal–Amorphous-Semiconductor Transition in Germanium. Phys. Rev. B: Condens. Matter Mater. Phys. 1994, 49, 14251–14269. 10.1103/PhysRevB.49.14251. 10010505

[ref77] Kresse G.; Furthmüller J. Efficiency of Ab-Initio Total Energy Calculations for Metals and Semiconductors Using a Plane-Wave Basis Set. Comput. Mater. Sci. 1996, 6, 15–50. 10.1016/0927-0256(96)00008-0. 9984901

[ref78] Kresse G.; Furthmüller J. Efficient Iterative Schemes for Ab Initio Total-Energy Calculations Using a Plane-Wave Basis Set. Phys. Rev. B: Condens. Matter Mater. Phys. 1996, 54, 11169–11186. 10.1103/PhysRevB.54.11169. 9984901

[ref79] Blöchl P. E. Projector Augmented-Wave Method. Phys. Rev. B: Condens. Matter Mater. Phys. 1994, 50, 17953–17979. 10.1103/PhysRevB.50.17953. 9976227

[ref80] Kresse G.; Joubert D. From Ultrasoft Pseudopotentials to the Projector Augmented-Wave Method. Phys. Rev. B: Condens. Matter Mater. Phys. 1999, 59, 1758–1775. 10.1103/PhysRevB.59.1758.

[ref81] Perdew J. P.; Burke K.; Ernzerhof M. Generalized Gradient Approximation Made Simple. Phys. Rev. Lett. 1996, 77, 3865–3868. 10.1103/PhysRevLett.77.3865. 10062328

[ref82] Anisimov V. I.; Aryasetiawan F.; Lichtenstein A. I. First-Principles Calculations of the Electronic Structure and Spectra of Strongly Correlated Systems: The LDA+ U Method. J. Phys. Condens. Matter 1997, 9, 767–808. 10.1088/0953-8984/9/4/002.

[ref83] Jain A.; Hautier G.; Ong S. P.; Moore C. J.; Fischer C. C.; Persson K. A.; Ceder G. Formation Enthalpies by Mixing GGA and GGA + U Calculations. Phys. Rev. B: Condens. Matter Mater. Phys. 2011, 84, 045115 10.1103/PhysRevB.84.045115.

[ref84] Wang L.; Maxisch T.; Ceder G. Oxidation Energies of Transition Metal Oxides within the GGA+ U Framework. Phys. Rev. B: Condens. Matter Mater. Phys. 2006, 73, 195107 10.1103/PhysRevB.73.195107.

[ref85] Ong S. P.; Richards W. D.; Jain A.; Hautier G.; Kocher M.; Cholia S.; Gunter D.; Chevrier V. L.; Persson K. A.; Ceder G. Python Materials Genomics (Pymatgen): A Robust, Open-Source Python Library for Materials Analysis. Comput. Mater. Sci. 2013, 68, 314–319. 10.1016/J.COMMATSCI.2012.10.028.

[ref86] Dovesi R.; Erba A.; Orlando R.; Zicovich-Wilson C. M.; Civalleri B.; Maschio L.; Rérat M.; Casassa S.; Baima J.; Salustro S.; Kirtman B. Quantum-Mechanical Condensed Matter Simulations with CRYSTAL. Wiley Interdiscip. Rev. Comput. Mol. Sci. 2018, 8, e1360 10.1002/wcms.1360.

[ref87] Becke A. D. A. New Mixing of Hartree-Fock and Local Density-Functional Theories. J. Chem. Phys. 1993, 98, 1372–1377. 10.1063/1.464304.

[ref88] Lee C.; Yang W.; Parr R. G. Development of the Colle-Salvetti Correlation-Energy Formula into a Functional of the Electron Density. Phys. Rev. B: Condens. Matter Mater. Phys. 1988, 37, 785–789. 10.1103/PhysRevB.37.785. 9944570

[ref89] Vosko S. H.; Wilk L.; Nusair M. Accurate Spin-Dependent Electron Liquid Correlation Energies for Local Spin Density Calculations: A Critical Analysis. Can. J. Phys. 1980, 58, 1200–1211. 10.1139/p80-159.

[ref90] Stephens P. J.; Devlin F. J.; Chabalowski C. F.; Frisch M. J. Ab Initio Calculation of Vibrational Absorption and Circular Dichroism Spectra Using Density Functional Force Fields. J. Phys. Chem. 1994, 98, 11623–11627. 10.1021/j100096a001.

[ref91] Corà F.; Alfredsson M.; Mallia G.; Middlemiss D. S.; Mackrodt W. C.; Dovesi R.; Orlando R. The Performance of Hybrid Density Functionals in Solid State Chemistry. Structure and Bonding; Springer: Berlin, Heidelberg, 2012; Vol. 113, pp 171–232.

[ref92] Muscat J.; Wander A.; Harrison N. M. On the Prediction of Band Gaps from Hybrid Functional Theory. Chem. Phys. Lett. 2001, 342, 397–401. 10.1016/S0009-2614(01)00616-9.

[ref93] Feng X.; Harrison N. M. Magnetic Coupling Constants from a Hybrid Density Functional with 35% Hartree-Fock Exchange. Phys. Rev. B: Condens. Matter Mater. Phys. 2004, 70, 092402 10.1103/PhysRevB.70.092402.

[ref94] Middlemiss D. S.; Lawton L. M.; Wilson C. C. A Solid-State Hybrid Density Functional Theory Study of Prussian Blue Analogues and Related Chlorides at Pressure. J. Phys. Condens. Matter 2008, 20, 335231 10.1088/0953-8984/20/33/335231.

[ref95] deMoreira P. R. I.; Illas F.; Martin R. L. Effect of Fock Exchange on the Electronic Structure and Magnetic Coupling in NiO. Phys. Rev. B: Condens. Matter Mater. Phys. 2002, 65, 155102 10.1103/PhysRevB.65.155102.

[ref96] Dovesi R.; Erba A.; Orlando R.; Zicovich-Wilson C. M.; Civalleri B.; Maschio L.; Rérat M.; Casassa S.; Baima J.; Salustro S.; Kirtman B. CRYSTAL17 User’s Manual; WIREs Comput. Mol. Sci., 2018; p e1360.

[ref97] Schäfer A.; Horn H.; Ahlrichs R. Fully Optimized Contracted Gaussian Basis Sets for Atoms Li to Kr. J. Chem. Phys. 1992, 97, 2571–2577. 10.1063/1.463096.

[ref98] Middlemiss D. S.; Ilott A. J.; Clément R. J.; Strobridge F. C.; Grey C. P. Density Functional Theory-Based Bond Pathway Decompositions of Hyperfine Shifts: Equipping Solid-State NMR to Characterize Atomic Environments in Paramagnetic Materials. Chem. Mater. 2013, 25, 1723–1734. 10.1021/cm400201t.

[ref99] Monkhorst H. J.; Pack J. D. Special Points for Brillouin-Zone Integrations. Phys. Rev. B: Solid State 1976, 13, 5188–5192. 10.1103/physrevb.13.5188.

