# Supplementary material for: Polymorphism in Weberite Na2Fe2F7 and its Effects on Electrochemical Properties as a Na-Ion Cathode
Source: Chem Mater. 2023 Apr 25;35(9):3614–27. doi: 10.1021/acs.chemmater.3c00233 (PMC10174150; doi:10.1021/acs.chemmater.3c00233)
Supplement: Supplementary file 1 — cm3c00233_si_001.pdf [file cm3c00233_si_001.pdf]

Supplementary Information for Polymorphism in Weberite $Na_2Fe_2F_7$ and its Effects on Electrochemical Properties as a Na-ion Cathode

Emily E. Foley,[1,2] Vincent C. Wu,[1,2] Wen Jin,[2,3] Wei Cui,[2,4] Eric Yoshida,†[1,2] Alexis Manche, ‡[1] Raphaële J. Clément[1,2*]

[1]Materials Department, University of California Santa Barbara, California 93106, USA

[2]Materials Research Laboratory, University of California Santa Barbara, California 93106, USA

[3]Chemical Engineering Department, University of California Santa Barbara, California 93106, USA

[4]Physics Department, University of California Santa Barbara, California 93106, USA

†Present address: Materials Department, University of California Irvine, California 92697, USA

‡Present address: School of Chemistry, University of St-Andrews, North Haugh, St-Andrews, KY16 9ST, UK

*Corresponding author email: rclement@ucsb.edu

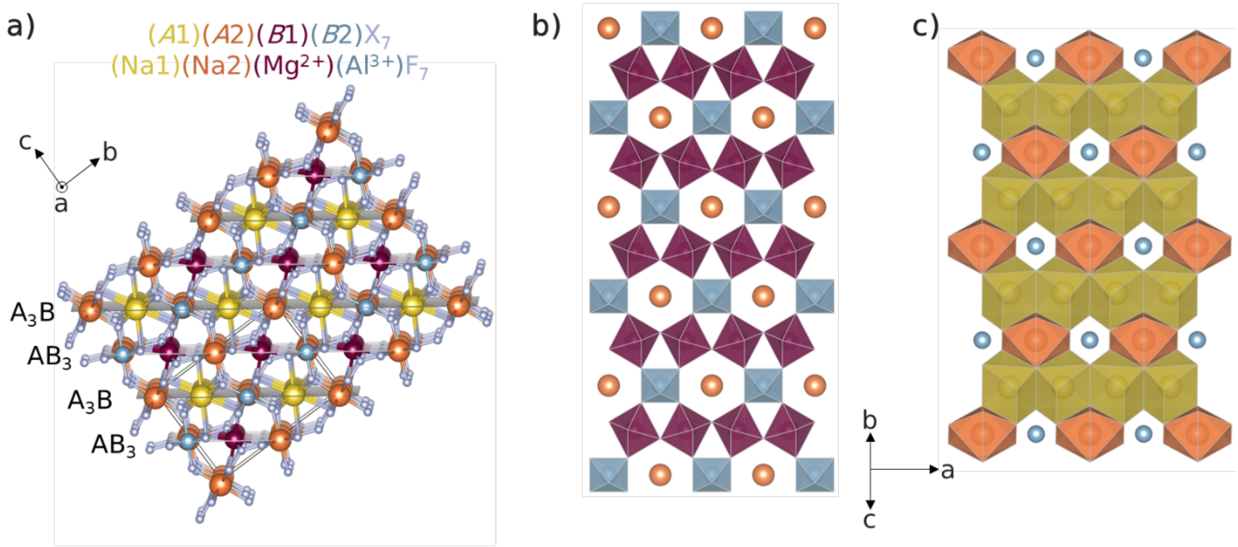

Figure S1. **a)** Stacking of the Kagomé-like (011) planes as viewed along the [100] direction in Na_2MgAlF_7. The unique Kagomé-like layers AB_3 and A_3B are shown in **b)** and **c)**, respectively, as viewed along the [011] direction.

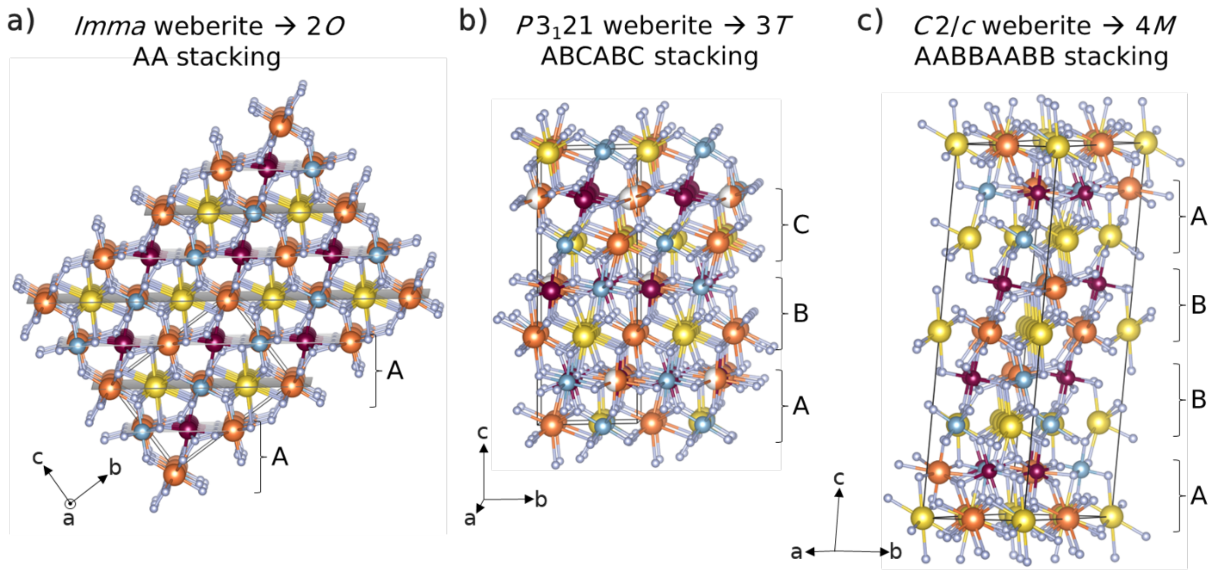

Figure S2. Crystal structure diagrams for the three weberite polymorphs: **a)** 2*O*, orthorhombic where all *B*1 chains are parallel to [100]; **b)** 3*T*, trigonal where *B*1 chains are parallel to [010], [110], and [100] for the *A*, *B*, *C* layers, respectively; **c)** and 4*M*, monoclinic where *B*1 chains are parallel to [$\bar{1}$10] and [110] for the *A* and *B* layers, respectively. Each slab (denoted by *A*, *B*, or *C*) corresponds to one AB_3 and one A_3B Kagomé-like layer. Note that within each *AA* and *BB* pair of the 4*M* structure, the A_3B layers differ slightly while the AB_3 layers remain identical.

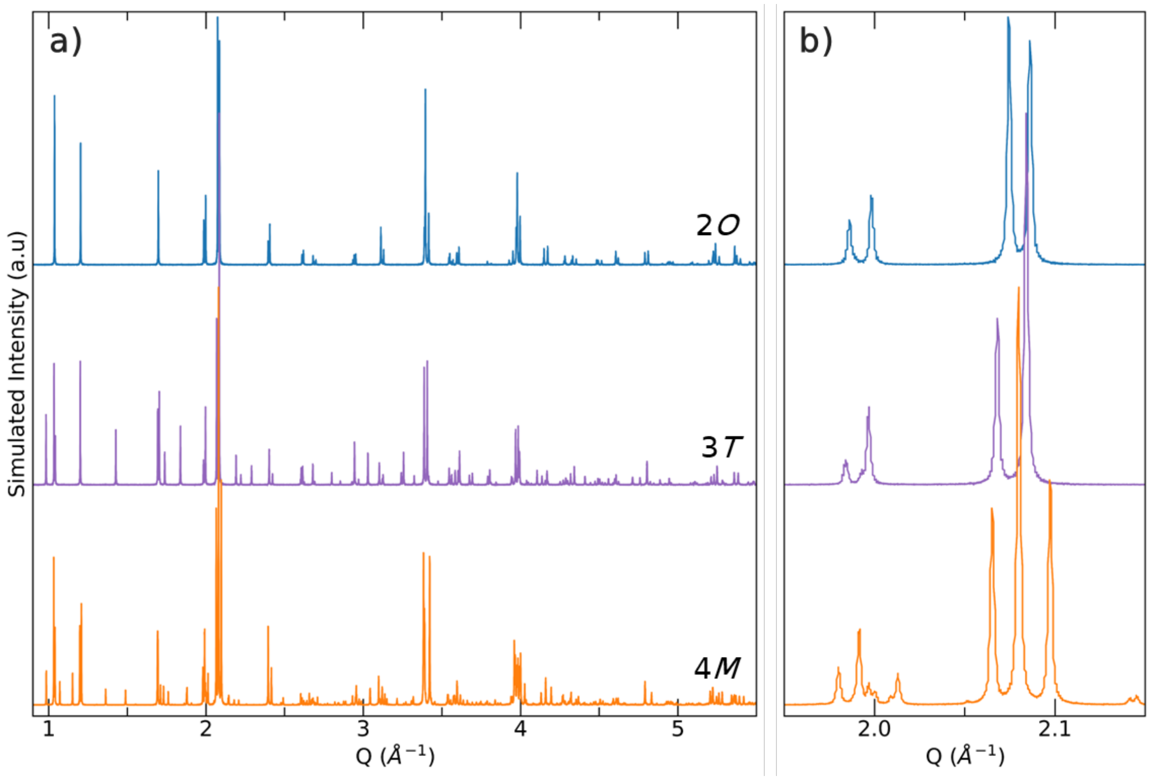

Figure S3. Simulated diffraction patterns for the three reported $Na_2Fe_2F_7$ weberite polymorphs: 2*O* (top), 3*T* (middle), and 4*M* (bottom). The full patterns are shown in **a)**, with an enlarged view of the highest intensity diffraction peaks shown in **b)**. Simulated diffraction patterns were obtained using GSAS-II and a step size of 0.001°.

Table S1. DFT-computed energy, formation energy (E_F), and energy above the hull (E_{Hull}) for all compounds within the NaF-FeF_2-FeF_3 phase space. The binary fluorides were used to compute the formation energies of the ternary phases.

	**Energy (meV/atom)**	**Energy (eV/f.u.)**	**E_F (meV/atom)**	**E_F (eV/f.u.)**	**E_{Hull} (meV/atom)**
**NaF**	−4324.62	−8.65	--	--	0.00
**FeF_2**	−5685.78	−17.06	--	--	0.00
**FeF_3**	−5317.44	−21.27	--	--	0.00
**$NaFeF_3$**	−5159.27	−25.80	−17.96	−0.09	0.00
**Na_3FeF_6**	−4809.44	−48.09	−87.69	−0.88	0.00
**$NaFeF_4$**	−4935.05	−108.57	−68.89	−1.52	7.68
**$Na_5Fe_3F_{14}$**	−5051.95	−30.31	−65.45	−0.39	0.00
**2*O*-$Na_2Fe_2F_7$**	−5108.21	−56.19	−51.34	−0.56	8.87
**3*T*-$Na_2Fe_2F_7$**	−5106.8	−56.17	−49.93	−0.55	10.28
**4*M*-$Na_2Fe_2F_7$**	−5105.58	−56.16	−48.71	−0.54	11.50

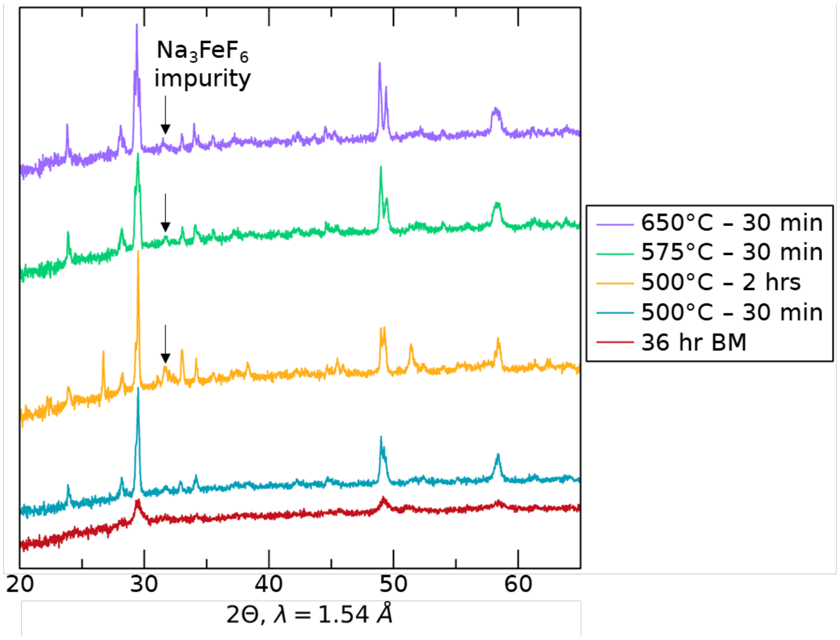

Figure S4. Laboratory XRD patterns of various synthesis conditions used to obtain $Na_2Fe_2F_7$. All syntheses began with a 36 hr ball-milling (BM) step (resulting XRD pattern shown in red) that was then followed by an anneal at the temperature and time specified in the figure. Na_3FeF_6 impurities were identified in all patterns besides the one obtained after a 500°C 30 min anneal, which was selected for the remainder of this work.

Table S2. Refined lattice constants and weight percentages for all phases used to fit the SXRD data collected on pristine $Na_2Fe_2F_7$. The fit residuals are: χ^2 = 2.12 and R_{WP} = 9.75%.

**Phase**	**2*O***	**3*T***	**4*M***	**Na_3FeF_6**	**FeF_2**
**Space Group**	*Imma*	*P*3₁21 → $P3_121$	*C*2/*c*	$P2_1$	$P4_2/mnm$
**a (Å)**	7.3811(4)	7.3726(1)	12.6985(3)	5.5195(2)	4.6932(1)
**b (Å)**	10.5049(6)	7.3726(1)	7.4094(2)	5.7166(2)	4.6932(1)
**c (Å)**	7.4377(3)	18.1752(4)	24.6541(6)	7.9621(3)	3.3162(1)
**alpha (°)**	90	90	90	90	90
**beta (°)**	90	90	100.132(1)	90.351(3)	90
**gamma (°)**	90	120	90	90	90
**wt%**	**3.0(2)%**	**41.2(4)%**	**45.6(4)%**	**9.4(1)%**	**0.90(2)%**

Table S3. Refined site parameters for all three weberite phases present in the pristine $Na_2Fe_2F_7$ sample.

**Phase**	**Atom**	**Wyckoff Position**	**x**	**y**	**z**	**Occ.**	**Beq**
2*O*	Na1	4*a*	0.000	0.000	0.000	1	1.16(8)
2*O*	Na2	4*d*	0.250	0.250	0.750	1	1.16(8)
2*O*	Fe1	4*c*	0.250	0.250	0.250	1	1.08(1)
2*O*	Fe2	4*b*	0.000	0.000	0.500	1	1.08(1)

2*O*	F1	4*e*	0.000	0.250	0.231(6)	1	1.20(6)
2*O*	F2	8*h*	0.000	0.401(3)	0.762(4)	1	1.20(6)
2*O*	F3	16*j*	0.187(3)	0.373(2)	0.404(3)	1	1.20(6)
3*T*	Na1	6*c*	0.5307(1)	0.873(1)	0.6713(6)	1	1.16(8)
3*T*	Na2	6*c*	0.82(3)	0.010(4)	0.3462(8)	0.5	1.16(8)
3*T*	Na3	6*c*	0.841(2)	0.014(3)	0.8381(9)	0.5	1.16(8)
3*T*	Fe1	3*a*	0.3364(9)	0.000	0.333	1	1.08(1)
3*T*	Fe2	3*b*	0.3263(7)	0.000	0.833	1	1.08(1)
3*T*	Fe3	6*c*	0.5037(6)	0.8368(7)	0.1688(2)	1	1.08(1)
3*T*	F1	6*c*	0.7456(1)	0.211(2)	0.6087(5)	1	1.20(6)
3*T*	F2	6*c*	0.748(1)	0.910(1)	0.9468(6)	1	1.20(6)
3*T*	F3	6*c*	0.544(2)	0.421(1)	0.0596(5)	1	1.20(6)
3*T*	F4	6*c*	0.832(2)	0.447(2)	0.1931(6)	1	1.20(6)
3*T*	F5	6*c*	0.026(2)	0.347(2)	0.2692(4)	1	1.20(6)
3*T*	F6	6*c*	0.203(2)	0.251(1)	0.1416(6)	1	1.20(6)
3*T*	F7	6*c*	0.069(2)	0.436(1)	0.8538(4)	1	1.20(6)
4*M*	Na1	4*a*	0.000	0.000	0.000	1	1.16(8)
4*M*	Na2	4*b*	0.000	0.500	0.000	1	1.16(8)
4*M*	Na3	4*d*	0.250	0.250	0.500	1	1.16(8)
4*M*	Na4	4*e*	0.000	0.758(2)	0.250	1	1.16(8)
4*M*	Na5	8*f*	0.2458(9)	0.0582(1)	0.2462(4)	1	1.16(8)
4*M*	Na6	8*f*	0.365(1)	0.501(2)	0.1245(4)	1	1.16(8)
4*M*	Fe1	4*c*	0.250	0.250	0.000	1	1.08(1)
4*M*	Fe2	4*e*	0.000	0.246	0.250	1	1.08(1)
4*M*	Fe3	8*f*	0.1299(4)	0.7578(6)	0.1291(2)	1	1.08(1)
4*M*	Fe4	8*f*	0.3775(5)	0.0132(8)	0.1306(2)	1	1.08(1)
4*M*	Fe5	8*f*	0.1236(5)	0.2613(6)	0.1244(3)	1	1.08(1)
4*M*	F1	8*f*	0.334(1)	0.054(1)	0.041(5)	1	1.20(6)
4*M*	F2	8*f*	0.422(1)	-0.044(2)	0.207(5)	1	1.20(6)
4*M*	F3	8*f*	0.464(1)	0.268(2)	0.125(5)	1	1.20(6)
4*M*	F4	8*f*	0.266(1)	-0.176(2)	0.100(5)	1	1.20(6)
4*M*	F5	8*f*	0.151(1)	0.3010(2)	0.047(6)	1	1.20(6)
4*M*	F6	8*f*	0.131(1)	0.305(2)	0.212(7)	1	1.20(6)
4*M*	F7	8*f*	0.055(1)	-0.002(2)	0.105(5)	1	1.20(6)
4*M*	F8	8*f*	0.163(1)	0.509(2)	0.141(5)	1	1.20(6)
4*M*	F9	8*f*	0.299(1)	0.212(2)	0.159(5)	1	1.20(6)
4*M*	F10	8*f*	-0.020(1)	0.334(2)	0.103(5)	1	1.20(6)
4*M*	F11	8*f*	0.321(1)	0.484(2)	0.032(5)	1	1.20(6)
4*M*	F12	8*f*	0.064(1)	0.065(2)	0.297(5)	1	1.20(6)
4*M*	F13	8*f*	0.155(1)	0.80(2)	0.202(7)	1	1.20(6)
4*M*	F14	8*f*	0.076(1)	0.703(2)	0.053(6)	1	1.20(6)

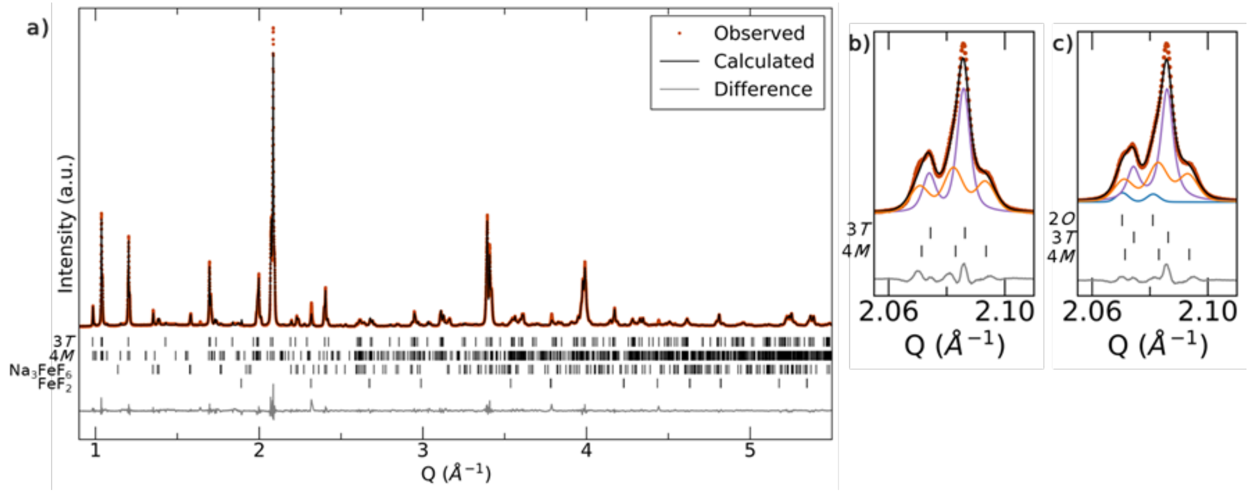

Figure S5. **a)** SXRD pattern collected on pristine $Na_2Fe_2F_7$ and refined using the Rietveld method and only the 3*T* and 4*M* weberite variants (χ^2 = 2.16, R_{WP} = 9.94%). An enlarged version of the refinement results of the main weberite peaks is shown in **b)**, and compared to the best refinement results (χ^2 = 2.12, R_{WP} = 9.75%) over the same Q range obtained using all three weberite polymorphs (2*O*, 3*T*, and 4*M*, see Figure 2a) in **c)**. Individual profiles are shown for the 2*O* (blue), 3*T* (purple), and 4*M* (orange) polymorphs.

Table S4. Refined lattice constants and weight percentages for all phases used to fit the SXRD data for carbon-coated $Na_2Fe_2F_7$. The fit residuals are: χ^2 = 1.31 and R_{WP} =7.01%. Note that the 4*M* weberite polymorph was used to fit the low crystallinity, carbon-coated data as it was the majority weberite variant in the pristine material.

	**4*M***	**Na_3FeF_6**
**Space Group**	*C*2/*c*	*P*2_1
**a (Å)**	12.749(2)	5.413(1)
**b (Å)**	7.381(1)	5.808(1)
**c (Å)**	24.452(6)	7.964(2)
**alpha (°)**	90	90
**beta (°)**	99.39(1)	90.646(6)
**gamma (°)**	90	90
**wt%**	**88.3(2)%**	**11.4(5)%**

Table S5. Fitting parameters for ^{57}Fe Mössbauer spectra collected on the pristine and carbon-coated $Na_2Fe_2F_7$ samples, and resulting average iron oxidation state and sodium stoichiometry in the weberite phase.

	**Signal**	**Isomer Shift (δ, mm/s)**	**Quadrupolar Splitting (ΔE_Q, mm/s)**	**FWHM (mm/s)**	**Integrated Intensity**	χ^2	**Avg. Fe Ox. State**		**Weberite Na Stoich.**
							**Weberite**	**Total**	
	Fe^{2+}- 1	1.45(6)	1.38(12)	0.43(3)	25(6)%				
	Fe^{2+}- 2	1.25(2)	1.42(3)	0.34(5)	13(5)%				
**Pristine**	Fe^{3+}- 1	0.43(4)	0.72(9)	0.39(4)	27(9)%	1.1	2.55(16)	2.62(15)	1.90(33)
	Fe^{3+}- 2	0.45(1)	0.49(4)	0.29(4)	20(8)%				
	$Na_3Fe^{3+}F_6$	0.27	0.15	0.68(9)	15(4)%				
	Fe^{2+}	1.41(9)	1.67(9)	0.94(8)	28(8)%				
**C-Coated**	Fe^{3+}	0.41(1)	0.73(3)	0.55(8)	54(9)%	1.2	2.66(15)	2.70(16)	1.68(30)
	$Na_3Fe^{3+}F_6$	0.27	0.15	0.68(9)	18(9)%				

Table S6. ICP results for $Na_2Fe_2F_7$. All data have been normalized to the Fe content. The errors listed here only account for uncertainties in the calibration curves and do not include other experimental errors, such as trace ions in the deionized water used for dilution and in the sample containers.

	**Na**	**Fe**
**Pristine**	1.94(1)	2
**Carbon-Coated**	1.95(1)	2

Supplemental Note 1. First principles computations of NMR parameters using CRYSTAL17 and comparison with experimental results

The computed hyperfine (paramagnetic) NMR properties were obtained at 0 K for the $Na_2Fe_2F_7$ 2*O*, 3*T*, and 4*M* weberite polymorphs using a 2x1x2 supercell for the 2*O* variant, and a 1x1x1 cell for the 3*T* and 4*M* variants. All computations were carried out on ferromagnetically-aligned cells. To compare CRYSTAL17 calculation results with experimental data acquired at room temperature, the computed shifts were subsequently scaled to a value consistent with the paramagnetic state of the system at the temperature of the NMR experiments, using a magnetic scaling factor Φ of the form:

$$\Phi(T_{exp}) = \frac{\langle M(T_{exp}) \rangle}{M_{sat}}, \qquad (1)$$

where M_{sat} is the saturated (ferromagnetic) $Fe^{2.5+}$ magnetic moment at 0 K, and $\langle M(T_{exp}) \rangle$ the bulk average magnetic moment measured at the sample experimental temperature, T_{exp}. Here, T_{exp} is set to 320 K to account for frictional heating caused by fast (60 kHz) sample rotation during NMR data acquisition.

The magnetic scaling factor in eq. (1) can be evaluated from the experimental magnetic properties of the material:

$$\Phi(\mathrm{T}) = \frac{B_0 \mu_{eff}{}^2}{3 k_B g_e \mu_B S (T - \Theta)}, \qquad (2)$$

where B_0 is the external magnetic field, μ_{eff} is the effective magnetic moment per Fe site, k_B is Boltzmann's constant, g_e is the free electron *g*-value, μ_B is the Bohr magneton, *S* is the formal spin of $Fe^{2.5+}$ ($S = 4.5/2$), and Θ is the Weiss constant. A derivation of eq. (2), starting from the Brillouin function in the low field, high temperature limit, can be found in a previous study by Kim et al.[1] Eq. (2) uses the "spin-only" expression for the magnetic moment and is only strictly valid when the orbital angular momentum is quenched.[2] Yet, for systems where spin-orbit coupling effects are negligible, such as $Na_2Fe_2F_7$, the spin-only expression is a good approximation of the magnetic behavior of the system.

Given the multi-phasic nature of $Na_2Fe_2F_7$ samples, we are unable to obtain the magnetic properties (μ_{eff} and Θ) of individual $Na_2Fe_2F_7$ polymorphs experimentally. However, similar magnetic properties may be expected for the different polymorphs as they all retain a similar transition metal network, solely differentiated by variations in layer stacking.[3] Thus, we approximated μ_{eff} by the theoretical, "spin-only" magnetic moment ($\mu_{SO} = 5.41\ \mu_B/Fe^{2.5+}$), and used a previously-reported value[3] of $\Theta = -104$ K for $Na_2Fe_2F_7$ to compute the magnetic scaling factor. A bulk magnetic scaling factor, Φ, of 0.008066 was obtained at T = 320 K and $B_0 = 2.35$ T using eq. (1), which was used to scale the computed ^{23}Na and parameters listed in **Table S7**.

Table S7. First principles ^{23}Na NMR parameters computed using the CRYSTAL17 code on 2O, 3T, and 4M $Na_2Fe_2F_7$ structures optimized with VASP. The predicted NMR properties were scaled using a scaling factor Φ = 0.008066 to compare them to room temperature ^{23}Na solid-state NMR data obtained at an external magnetic field of B_0 = 2.35 T. There are two unique Na local environments in 2*O*, three in 3*T*, and six in 4*M* $Na_2Fe_2F_7$, with multiplicities specified in parentheses in the table below. δ_{iso} is the isotropic Fermi contact shift, $\Delta\delta$ and η are the electron-nuclear dipolar anisotropy and asymmetry parameters, respectively, C_Q is the quadrupolar coupling constant, η_Q is the quadrupolar asymmetry, δ_Q is the second-order quadrupolar shift, and $\delta_{obs} = \delta_{iso} + \delta_Q$ is the observed chemical shift.

Phase	Site	Coord. Type	δ_{iso} (ppm)	$\Delta\delta$ (ppm)	η	$\vert QCC \vert$ (MHz)	η_Q	δ_Q (ppm)	δ_{iso} - δ_Q (ppm)
**2O**	**Na1 (x1)**	cubic	218	207300	0.87	3.53	0.48	479	-261
**2O**	**Na2 (x1)**	bihex.	347	-246700	0.65	6.24	0.61	1562	-1215
**3T**	**Na1 (x2)**	cubic	650	-2191	0.48	2.73	0.65	314	336
**3T**	**Na2 (x1)**	cubic	494	-2028	0.17	1.31	0.89	78	416
**3T**	**Na3 (x1)**	bihex.	694	-2133	0.50	5.62	0.34	1169	-475
**4M**	**Na1 (x1)**	cubic	505	-2124	0.78	2.61	0.67	280	224
**4M**	**Na2 (x1)**	bihex.	801	-2243	0.45	5.00	0.37	934	-133
**4M**	**Na3 (x1)**	cubic	516	-2028	0.83	2.62	0.36	256	260
**4M**	**Na4 (x1)**	cubic	529	-1974	0.09	0.89	0.66	33	497
**4M**	**Na5 (x2)**	cubic	680	-2238	0.47	2.80	0.40	296	384
**4M**	**Na6 (x2)**	bihex.	673	-2181	0.44	5.77	0.45	1267	-593

Table S8. Unit cell parameters for the reported experimental $Na_2Fe_2F_7$ structures[4–6] and for the structures optimized using the H20 and H35 functionals in this work.

	**2*O***			**3*T***			**4*M***		
	**Exp.**	**Opt. H20**	**Opt. H35**	**Exp.**	**Opt. H20**	**Opt. H35**	**Exp.**	**Opt. H20**	**Opt. H35**
**a (Å)**	7.357	7.449	7.475	7.377	7.374	7.359	12.767	12.646	12.604
**b (Å)**	10.492	10.266	10.275	7.377	7.366	7.358	7.422	7.423	7.381
**c (Å)**	7.418	7.479	7.336	18.229	18.202	18.075	24.710	24.740	24.645
**alpha (°)**	90	90	90	90	90.054	90	90	90	90
**beta (°)**	90	90	90	90	90.149	90	99.970	99.710	99.815
**gamma (°)**	90	90	90	120	119.675	120	90	90	90
**Volume ($Å^3$)**	572.569	571.939	563.484	859.202	859.051	847.570	2341.437	2289.023	2259.164

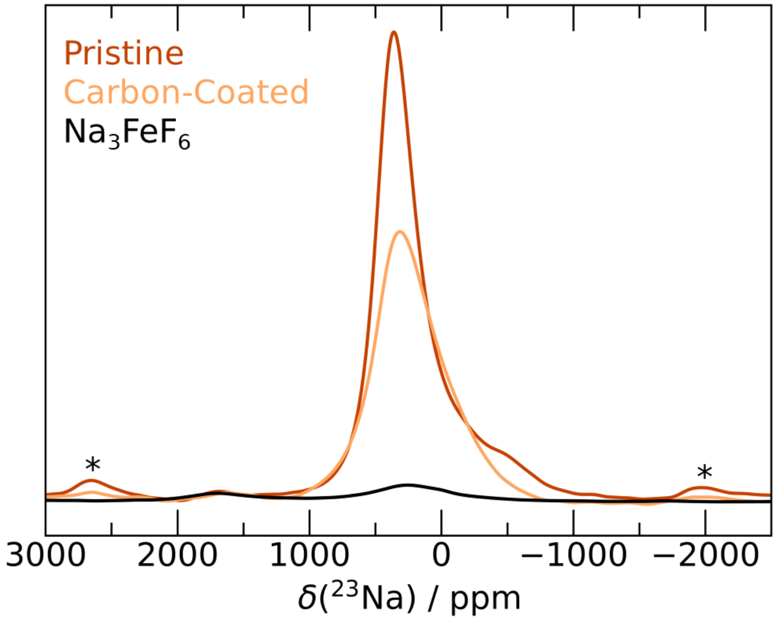

Figure S6. Comparison of ^{23}Na solid-state NMR spin echo spectra collected with a π/2 excitation pulse on Na_3FeF_6, and on pristine and carbon-coated $Na_2Fe_2F_7$. All spectra are scaled according to the intensity of the 1800 ppm Na_3FeF_6 signal. Asterisks indicate spinning sidebands.

Supplementary Note 2. Optimization of the carbon-coating method for the preparation of $Na_2Fe_2F_7$ cathode films

As the 24 hr carbon-coating process affects the weberite structure (based on the SXRD and Mössbauer results in **Figure 2-3**, and ^{23}Na ss-NMR results presented in **Figure 4** in the main text), several shorter mechanochemical milling times and an *in situ* carbon-coating method were investigated to reduce structural disordering. The *in situ* carbon-coating method involved mixing the $Na_2Fe_2F_7$ material with sugar prior to the annealing step and then heating the mixture to 650°C for 30 min. The laboratory XRD patterns collected on the various cathode films are shown in **Figure S7a**. These cathode films were galvanostatically cycled at a rate of C/20 (full (dis)charge in 20 hrs assuming the transfer of 2 Na per formula unit) by first charging to 4.3 V vs. Na^+/Na and subsequent cycling between 4.3 V and 1.5 V. The resulting electrochemical profiles are shown in **Figure S7b**. While the *in situ* carbon-coating method led to a highly crystalline cathode, it also resulted in significant decomposition of the weberite phases. Further, the shorter milling procedures all resulted in reduced crystallinity and worse electrochemistry than the 24 hr ball-milled carbon-coated $Na_2Fe_2F_7$ cathode considered thus far. Hence, carbon-coating using a 24 hr ball-milling step was deemed optimal and used for the remainder of this study.

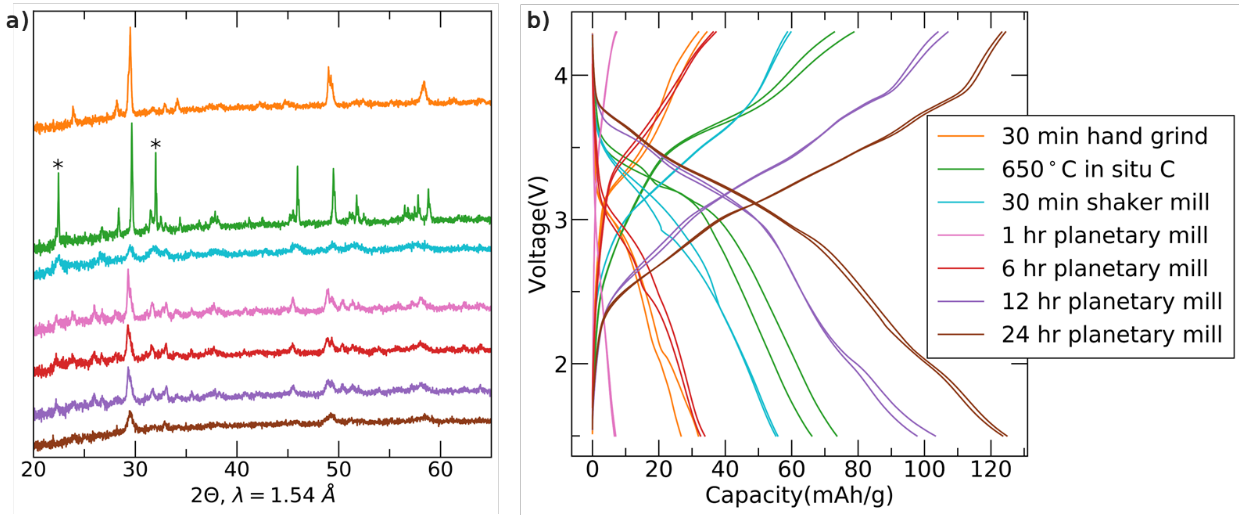

Figure S7. **a)** Laboratory XRD patterns, and **b)** galvanostatic charge-discharge curves obtained for $Na_2Fe_2F_7$ after different carbon-coating methods. Asterisks denote impurity/decomposition phases formed during the carbon-coating step.

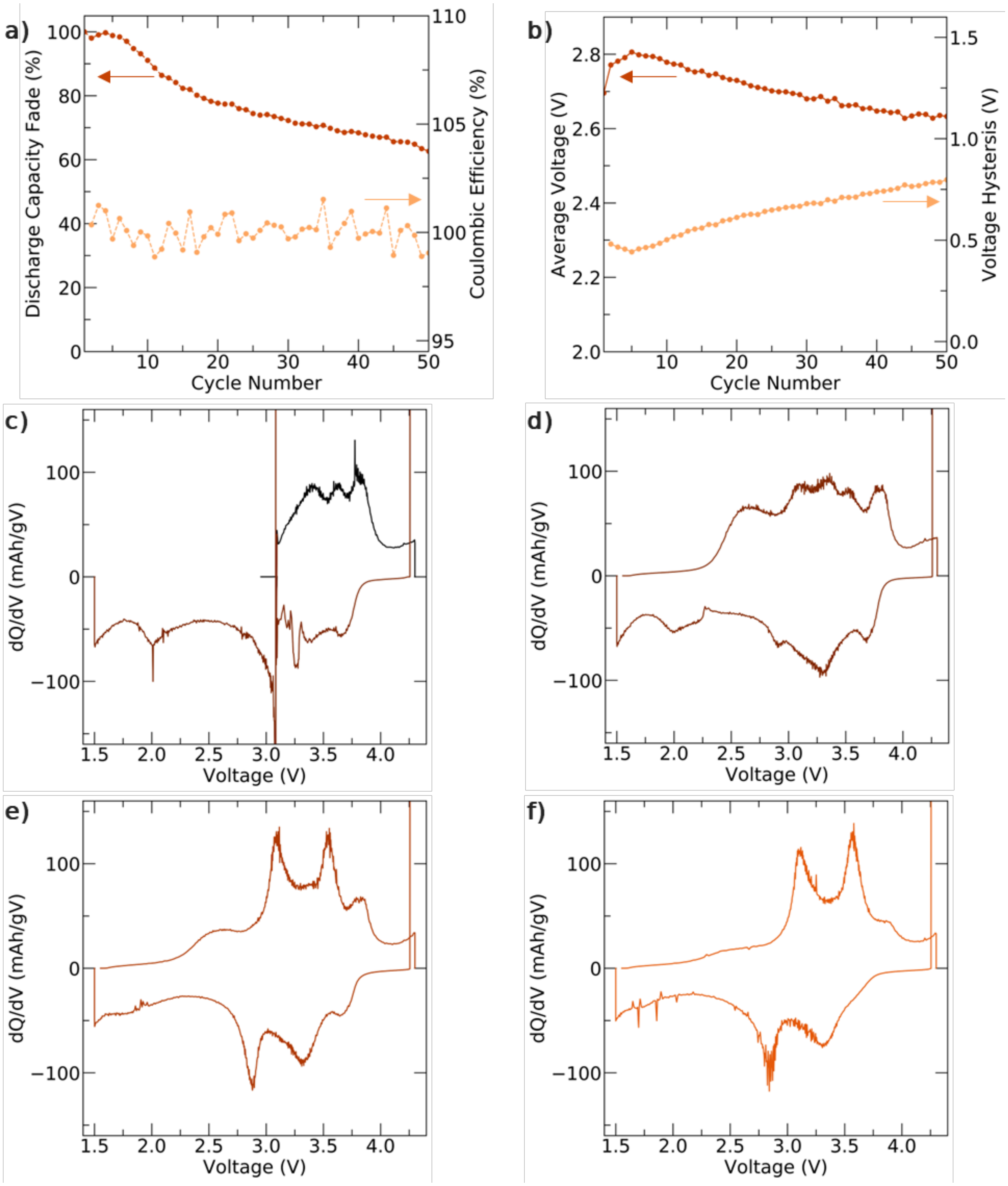

Figure S8. **a)** Plots of discharge capacity retention and coulombic efficiency and **b)** average discharge voltage and voltage hysteresis vs. cycle number for the galvanostatic data shown in **Figure 5**. dQ/dV plots for cycles 1 **(c)**, 2 **(d)**, 10 **(e)**, and 20 **(f)** from the galvanostatic data shown in **Figure 5b**. The cycle 1 charge data is shown in black with the remaining data coloring analogous to that in **Figure 5b**.

Table S9. Refined lattice constants and weight percentages for all phases used to fit the SXRD data collected on *ex situ* 1st discharge $Na_2Fe_2F_7$. The fit residuals are: χ^2 = 1.18 and R_{WP} =4.84%.

**4*M***	**Na_3FeF_6**	**$NaFeF_3$**
*C*2/*c*	*P*2$_1$	*Pnma*
12.533(3)	5.508(2)	5.679(2)
7.390(2)	5.702(2	7.917(2)
24.497(6)	8.003(2)	5.499(1)
90	90	90
99.94(1)	90.646(6)	90
90	90	90
**54.4(3)%**	**4.9(4)%**	**40.8(3)%**

Table S10. Refined lattice constants and weight percentages for all phases used to fit the SXRD data collected on *ex situ* 10th discharge $Na_2Fe_2F_7$. The fit residuals are: χ^2 = 1.19 and R_{WP} = 4.98%.

**4*M***	**Na_3FeF_6**	**$NaFeF_3$**
*C*2/*c*	*P*2$_1$	*Pnma*
12.624(2)	5.493(2)	5.6725(9)
7.377(1)	5.697(1)	7.899(1)
24.577(4)	7.980(2)	5.4917(9)
90	90	90
100.506(6)	90.53(2)	90
90	90	90
**37.6(3)%**	**9.2(4)%**	**53.2(4)%**

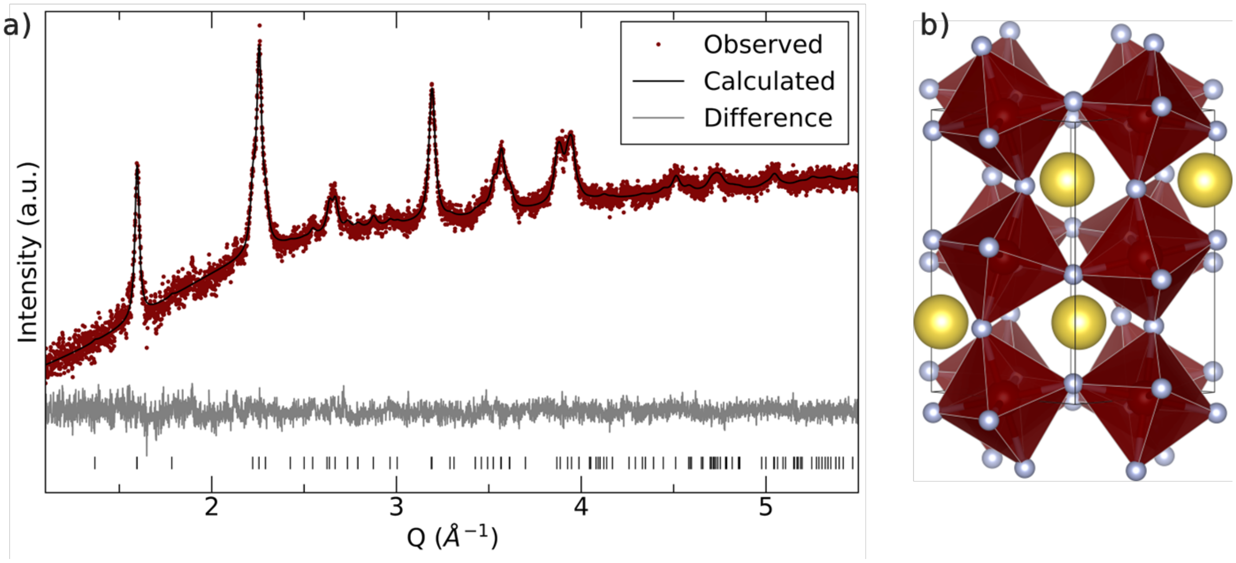

Figure S9. **a)** Laboratory XRD pattern collected on $NaFeF_3$ prepared via 12 hrs of ball-milling at 600 rpm and corresponding Rietveld refinement. Refined lattice parameters are *a* = 5.48782, *b* = 7.88543, *c* = 5.65752, with a χ^2 = 1.54. **b)** Perovskite $NaFeF_3$ (*Pnma*) structure viewed along the [101] direction.

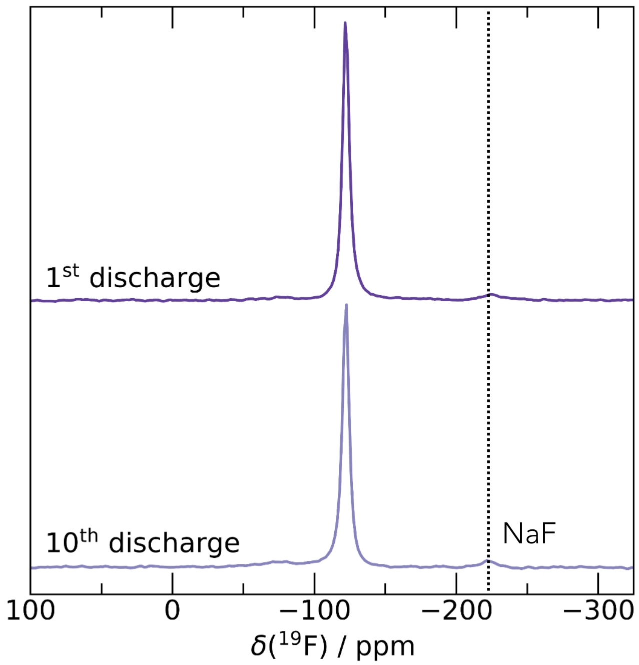

Figure S10. Quantitative ^{19}F ss-NMR spin echo spectra obtained on *ex situ* $Na_xFe_2F_7$ samples collected after the 1st and 10th discharge using a long interpulse delay of 60 s. Both spectra show a prominent signal at −120 ppm assigned to the PTFE binder, and very minor signals at −74.5 ppm and −224 ppm, which we attribute to decomposed electrolyte species containing PF_6^-,[7] and NaF, respectively. The NaF signal is indicated with a dashed line. By taking the ratio of NaF to PTFE integrated signal intensity (assuming the presence of 10 wt% PTFE in the sample, as used to prepare the cathode films), we find that the NaF content in the two *ex situ* samples is < 0.05 wt%. Thus, NaF is not related to the phase transformation process occurring in the bulk of the cathode and most likely arises from electrolyte decomposition during cycling. We note that NMR signals from ^{19}F nuclei directly bonded to paramagnetic Fe species (as is the case for all F environments in $Na_xFe_2F_7$ and Na_yFeF_3) in the cathode materials are too broad (and short-lived) to be observed experimentally.

Supplemental Note 3. Selection of Na-vacancy enumerated structures

All calculations were performed on 1x1x1 cells of the different weberite variants, leading to cells containing 44, 66, and 176 atoms for 2*O*, 3*T*, and 4*M* $Na_2Fe_2F_7$, respectively. As each weberite polymorph contains many individual Na sites, it is impractical to consider all possible Na-vacancy orderings. For example, there are 17,000 Na-vacancy orderings between $x = 1$ and 2 for the 4*M* polymorph. Thus, select configurations were considered for each polymorph at x = 0, 0.75, 1, 1.25, 1.5, 1.75, 2, 2.25, 2.5, and 3, and for the 4*M* polymorph only full or zero occupation of each of the Wyckoff sites was considered. Symmetrically-unique Na-vacancy orderings were enumerated and ranked according to their Ewald Sum Energy using Pymatgen (Python Materials Genomics)[8] and the five lowest energy orderings were considered. In total, 71 $Na_xFe_2F_7$ structures were calculated for the 2*O* polymorph, 12 for the 3*T* polymorph, and 85 for the 4*M* polymorph. Only a small number of structures were calculated for the 3T $Na_xFe_2F_7$ polymorph as a convex hull has already been reported for this variant, although only down to $x = 0.5$.[5]

As the 2*O* and 4*M* polymorphs contain no intrinsic Na vacancies, bond valence sum mapping using the SoftBV[9–11] software program was used to identify possible intercalation sites. This resulted in three and five possible intercalation sites for the 2*O* and 4*M* polymorphs, respectively. The coordinates for these possible intercalation sites are listed in **Table S11**. As the 3*T* structure contains two half-filled Na sites at $x = 2$, no additional intercalation sites were investigated. Below $x = 2$, only the Na sites that are occupied at $x = 2$ were considered.

Table S11. Possible Na-ion intercalation sites in 2O, 3T, and 4M $Na_xFe_2F_7$. Crystallographic information for sites that may be able to accommodate Na-ions in the various polymorphs, including sites occupied in the $Na_2Fe_2F_7$ crystal structures and those predicted by SoftBV.[9–11] The occupancy (occ.) is given at $x = 2$. The relative site energy is calculated using bond valence sum mapping within SoftBV. The coordination environment for each site is also provided, including the number of nearest neighbors (NN) and coordination environment.

Phase	Site	Occ.	Wyckoff Position	Coordinates			Relative Site Energy (eV/atom)	Coordination	
				x	y	z		NN	Polyhedron-Type
**2*O***	**Na1**	1	4*a*	0	0	0	0	8	Bihexagonal Pyramidal
**2*O***	**Na2**	1	4*d*	0.25	0.25	0.75	0.085	8	Cubic
**2*O***	***i*1**	0	4*e*	0.5	0.25	0.917	0.491	6	Prismatic
**2*O***	***i*2**	0	8*g*	0.75	0.542	0.25	0.562	6	Prismatic
**2*O***	***i*3**	0	16*j*	0.24	0.558	0.208	0.569	6	Prismatic
**3*T***	**Na1**	1	6*c*	0.523	0.849	0.665	0	8	Cubic
**3*T***	**Na2**	0.5	6*c*	0.949	0.186	0.336	0.019	8	Cubic
**3*T***	**Na3**	0.5	6*c*	0.902	0.169	0.863	0.014	8	Bihexagonal Pyramidal
**4*M***	**Na1**	1	4*a*	0	0	0.5	0	8	Cubic
**4*M***	**Na2**	1	4*b*	0.5	0	0	0.019	8	Bihexagonal Pyramidal
**4*M***	**Na5**	1	4*d*	0.736	0.042	0.25	0.014	8	Cubic
**4*M***	**Na3**	1	4*e*	0.25	0.25	0.5	0.043	8	Cubic
**4*M***	**Na4**	1	8*f*	0.5	0.729	0.75	0.048	8	Cubic
**4*M***	**Na6**	1	8*f*	0.861	0.979	0.125	0.112	8	Bihexagonal Pyramidal
**4*M***	***i*1**	0	8*f*	0.333	0.458	0.458	0.578	6	Prismatic
**4*M***	***i*2**	0	8*f*	0.208	0.438	0.556	0.611	6	Prismatic
**4*M***	***i*3**	0	8*f*	0.458	0.667	0.563	0.699	6	Prismatic
**4*M***	***i*4**	0	8*f*	0.306	0.271	0.701	0.725	6	Prismatic
**4*M***	***i*5**	0	8*f*	0.056	0.979	0.194	0.736	6	Prismatic

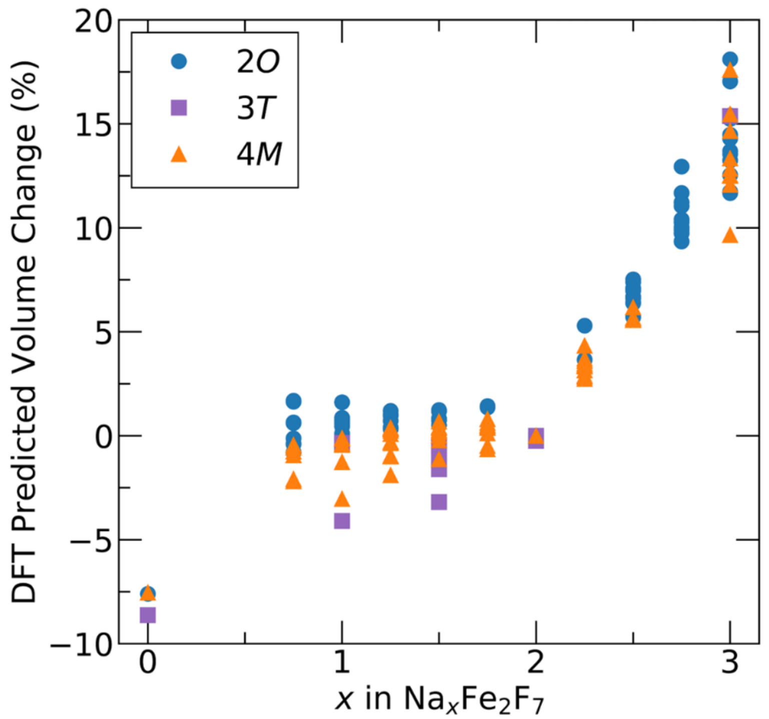

Figure S11. Predicted % volume change with respect to the $Na_2Fe_2F_7$ structure upon Na insertion and extraction for the three $Na_2Fe_2F_7$ weberite polymorphs. The volume expansion is plotted for each Na-vacancy ordering within 10 meV/atom of the lowest energy ordering at a given $Na_xFe_2F_7$ composition.

References

1 J. Kim, D. S. Middlemiss, N. A. Chernova, B. Y. X. Zhu, C. Masquelier and C. P. Grey, Linking local environments and hyperfine shifts: A combined experimental and theoretical 31P and 7Li solid-state NMR study of paramagnetic Fe(III) phosphates, *J. Am. Chem. Soc.*, 2010, **132**, 16825–16840.

2 C. Delmas, D. Carlier, G. Ceder, M. Ménétrier and C. P. Grey, Understanding the NMR shifts in paramagnetic transition metal oxides using density functional theory calculations, *Phys. Rev. B*, 2003, **67**, 174103.

3 A. Tressaud, J. M. Dance, J. Portier and P. Hagenmuller, Interactions magnetiques dans les fluorures de type weberite, *Mat. Res. Bull*, 1974, **9**, 1219–1226.

4 U. K. Dey, N. Barman, S. Ghosh, S. Sarkar, S. C. Peter and P. Senguttuvan, Topochemical Bottom-Up Synthesis of 2D- and 3D-Sodium Iron Fluoride Frameworks, *Chem. Mater.*, 2019, **31**, 295–299.

5 H. Park, Y. Lee, M.-K. Cho, J. Kang, W. Ko, Y. H. Jung, T.-Y. Jeon, J. Hong, H. Kim, S.-T. Myung and J. Kim, Na2Fe2F7: a fluoride-based cathode for high power and long life Na-ion batteries, *Energy Environ. Sci*, 2021, **14**, 1469–1479.

6 O. Yakubovich, V. Urusov, W. Massa, G. Frenzen and D. Babel, Structure of Na2Fe2F7 and structural relations in the family of weberites Na2MIIMIIIF7, *Zeitschrift für Anorg. und Allg. Chemie*, 1993, **619**, 1909–1919.

7 N. Dupre, M. Cuisinier and D. Guyomard, Electrode/Electrolyte Interface Studies in Lithium Batteries Using NMR, *Interface Mag.*, 2011, **20**, 61–67.

8 S. P. Ong, W. D. Richards, A. Jain, G. Hautier, M. Kocher, S. Cholia, D. Gunter, V. L. Chevrier, K. A. Persson and G. Ceder, Python Materials Genomics (pymatgen): A robust, open-source python library for materials analysis, *Comput. Mater. Sci.*, 2013, **68**, 314–319.

9 H. Chen and S. Adams, Bond softness sensitive bond-valence parameters for crystal structure plausibility tests, *IUCrJ*, 2017, **4**, 614–625.

10 H. Chen, L. L. Wong and S. Adams, SoftBV – a software tool for screening the materials genome of inorganic fast ion conductors, *Acta Crystallogr. Sect. B Struct. Sci.*, 2019, **75**, 18–33.

11 L. L. Wong, K. C. Phuah, R. Dai, H. Chen, W. S. Chew and S. Adams, Bond Valence Pathway Analyzer-An Automatic Rapid Screening Tool for Fast Ion Conductors within softBV, *Chem. Mater.*, 2021, **33**, 625–641.
